# JTK: targeted diploid genome assembler

**DOI:** 10.1093/bioinformatics/btad398

**Published:** 2023-06-24

**Authors:** Bansho Masutani, Yoshihiko Suzuki, Yuta Suzuki, Shinichi Morishita

**Affiliations:** Department of Computational Biology and Medical Sciences, Graduate School of Frontier Sciences, The University of Tokyo, Chiba 277-8562, Japan; Department of Computational Biology and Medical Sciences, Graduate School of Frontier Sciences, The University of Tokyo, Chiba 277-8562, Japan; Department of Computational Biology and Medical Sciences, Graduate School of Frontier Sciences, The University of Tokyo, Chiba 277-8562, Japan; Department of Computational Biology and Medical Sciences, Graduate School of Frontier Sciences, The University of Tokyo, Chiba 277-8562, Japan

## Abstract

**Motivation:**

Diploid assembly, or determining sequences of homologous chromosomes separately, is essential to elucidate genetic differences between haplotypes. One approach is to call and phase single nucleotide variants (SNVs) on a reference sequence. However, this approach becomes unstable on large segmental duplications (SDs) or structural variations (SVs) because the alignments of reads deriving from these regions tend to be unreliable. Another approach is to use highly accurate PacBio HiFi reads to output diploid assembly directly. Nonetheless, HiFi reads cannot phase homozygous regions longer than their length and require oxford nanopore technology (ONT) reads or Hi-C to produce a fully phased assembly. Is a single long-read sequencing technology sufficient to create an accurate diploid assembly?

**Results:**

Here, we present JTK, a megabase-scale diploid genome assembler. It first randomly samples kilobase-scale sequences (called ‘chunks’) from the long reads, phases variants found on them, and produces two haplotypes. The novel idea of JTK is to utilize chunks to capture SNVs and SVs simultaneously. From 60-fold ONT reads on the HG002 and a Japanese sample, it fully assembled two haplotypes with approximately 99.9% accuracy on the histocompatibility complex (MHC) and the leukocyte receptor complex (LRC) regions, which was impossible by the reference-based approach. In addition, in the LRC region on a Japanese sample, JTK output an assembly of better contiguity than those built from high-coverage HiFi+Hi-C. In the coming age of pan-genomics, JTK would complement the reference-based phasing method to assemble the difficult-to-assemble but medically important regions.

**Availability and implementation:**

JTK is available at https://github.com/ban-m/jtk, and the datasets are available at https://doi.org/10.5281/zenodo.7790310 or JGAS000580 in DDBJ.

## 1 Introduction

In human genomes, the differences between homologous chromosomes are medically important in cases such as the major histocompatibility complex (MHC) and the leukocyte receptor complex (LRC) (Houwaart *et al.* 2022). For example, genome-wide association studies have frequently reported variants in these regions as causal (Lenz *et al.* 2016). Also, genes in these regions affect the inflammation risk in bone marrow transplantation (Morishima *et al.* 2010). We need haplotype information in these cases due to high linkage disequilibrium and the large differences between haplotypes (Trowsdale 2005).

In addition, it is desirable to assemble diploid genomes at a population scale with minimal manual curation to understand how many variants we have missed (Jarvis *et al.* 2022). For example, a recent study sequenced 50 parent–child trios from a population, phased 100 MHC haplotypes, and found many novel structural variants (SVs) (Jensen *et al.* 2017). However, since these assemblies were built with short-read sequencers, they could use only 50 haplotypes out of 200 sequenced haplotypes (=four haplotypes per one trio). Thus, to further investigate the diversity among a population, we need an approach for the accurate diploid genomes assembly.

One approach to obtain a diploid assembly is to use a reference genome or assemble primary contigs, find heterozygous SNVs and SVs, phase these variants, and determine phased blocks (Garg *et al.* 2021). DeepVariant (Poplin *et al.* 2018) and cuteSV (Jiang *et al.* 2020) are popular software to call variants, and LongPhase (Lin *et al.* 2022) is the latest software that phases SVs and SNVs. As long as we can align the reads accurately, variant calling and phasing methods are robust to sequencing errors, and this approach correctly phases most of the variants.

**Figure 1.**
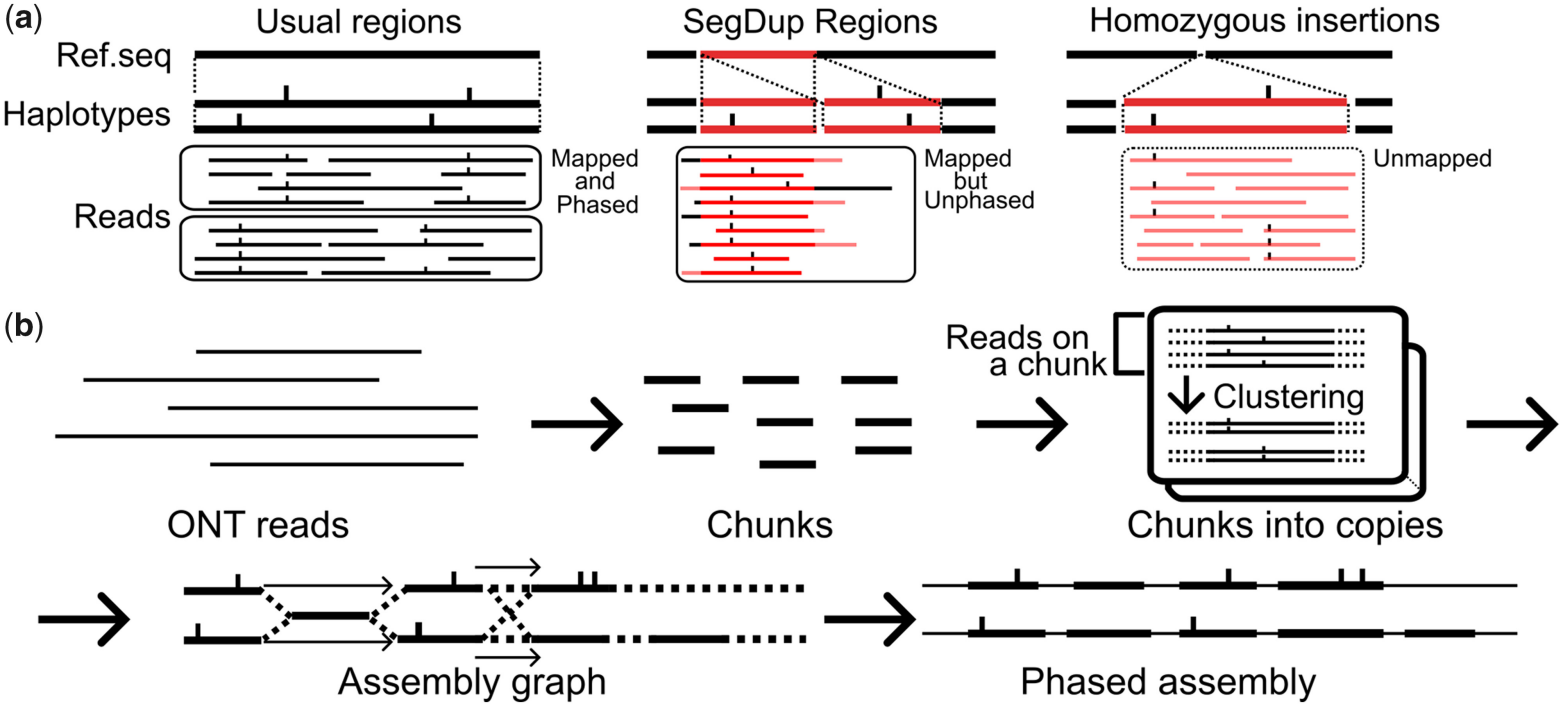
(a) The issues of the reference-based phasing. The short ticks are the variants on the reference or reads. Left: when the reference and two haplotypes are well correlated, we can map reads, find variants, and phase these reads. Center: when the reference underrepresents SDs, the alignments mix up these SDs, and phasing becomes inaccurate. Right: when the reference has the SVs, we cannot align the reads, and these reads remain unmapped and unphased. (b) The overview of JTK. It randomly samples chunks from the ONT reads. Then, JTK maps these chunks to the reads and separates the chunks into their copies. Next, it constructs a partially phased assembly graph and resolves the remaining regions to get a fully phased assembly.

However, this approach fails when the alignments of the reads are unreliable, and thus the variant calling is challenging, especially in the following two cases. One case is when the genome of a sample contains large segmental duplications (SDs) that are not represented in the reference (Fig. 1a, center). The other case is when two haplotypes contain large SVs compared to the reference (Fig. 1a, right).

Another approach is to use PacBio’s HiFi reads. Their high accuracy (>99.9%) enables us to accurately reconstruct diploid contigs directly. Hi-Canu (Nurk *et al.* 2020), HiFiASM (Cheng *et al.* 2021, 2022), and Verkko (Rautiainen *et al.* 2022) are software in this group. Since this approach does not depend on reference sequences, it is free from the issues in the phasing-based approach.

Nonetheless, to distinguish haplotypes in highly homozygous regions longer than HiFi reads (up to 30 kb), this approach needs other data such as Hi-C, Illumina’s trio data, strand-seq, or ONTs ultralong reads (Cheng *et al.* 2022, Rautiainen *et al.* 2022). For example, Verkko recommends using 50-fold HiFi reads, 50-fold ONT reads longer than 100 kb, and 50x of parental Illumina reads or Hi-C reads to achieve a nearly complete diploid assembly. These requirements can be a budget bottleneck when we try to represent human genetic diversity by assembling diploid genomes on a population scale.

To overcome these problems, some software packages such as PECAT and Phasebook attempt to construct diploid genomes from long ONT reads. However, these approaches correct errors in ONT reads by aligning them with other ONT reads, which can lead to overcollections and loss of informative SNVs and the inability to separate homologous chromosomes.

Our proposed software, JTK, produces the diploid assembly of the hard-to-assemble region in the genome, such as MHC and KIR, with a single sequencing technique (60-fold ONT reads). It takes three steps to assemble nearly identical regions and large SVs simultaneously. (i) JTK samples kilobase-scale subsequences (chunks) from the reads and aligns these chunks to the reads. By using a relaxed similarity threshold, a chunk can represent similar sequences (copies) of the chunk. (ii) JTK finds SNVs on these chunks and separates the chunks into each copy. To exhaustively enumerate SNVs on a chunk, JTK introduces each possible SNV to the chunk and accepts it as an actual SNV if the alignment scores of many reads increase. (iii) JTK determines the order of these separated copies in the target region and constructs a graph based on these orderings. Then, it produces the assembly by traversing the graph.

Evaluation of JTK with two simulated and six real datasets demonstrated that JTK provides fully resolved diploid assemblies from 60-fold ONT reads, which was impossible by a reference-based method and other ONT-only approaches. In addition, our assemblies had high base-level concordance to the assemblies based on 100-fold HiFi reads, 85-fold ONT reads longer than 100 kb, and Hi-C reads.

## 2 Materials and methods

### 2.1 Overview

As ONT reads contain 5%–10% errors, it is hard to construct the diploid assembly of the target region based on overlaps between reads. Also, since two haplotypes in the target region contain significant variations, such as SDs and SVs, it is challenging to construct a haploid assembly by squishing the variants between haplotypes.

To avoid these issues, JTK first samples subsequences (chunks) from the ONT reads (reads) (Fig. 1b). Each chunk represents similar sequences in the underlying target region, such as repeats with high similarity and homologous sequences, which we call “copies” of the chunk. By selecting chunks so that they cover the target region uniformly, we represent the target region by these chunks. Then, JTK decompose chunks into their copies, determines the order of them in the target region, and constructs a partially phased assembly graph. Finally, it produces a fully phased assembly by leveraging the ONT reads (Fig. 1b).

While traditional assemblers rebuild the genome from overlaps between reads, JTK assembles the genome gradually from chunks. Using these chunks, JTK can capture heterozygous insertions and deletions by the presence or absence of chunks from these SVs. JTK also captures SNVs between haplotypes by finding SNVs on the chunks.

### 2.2 Defining the chunks and the copy numbers of them

We randomly sample *L*-bp chunks from the reads to cover the target region with 1-fold coverage. We will discuss how to determine *L* later in this section, but shortly, we use L=2000 by default. Then, we align these chunks to the reads allowing errors up to 20% so that regions similar to a chunk are represented as the same chunk. We refer to these regions as “copies” of the chunk and denote the number of these regions as the “copy number” of the chunk. As we will separate the chunks into their copies, it is essential to accurately estimate these copy numbers.

**Figure 2.**
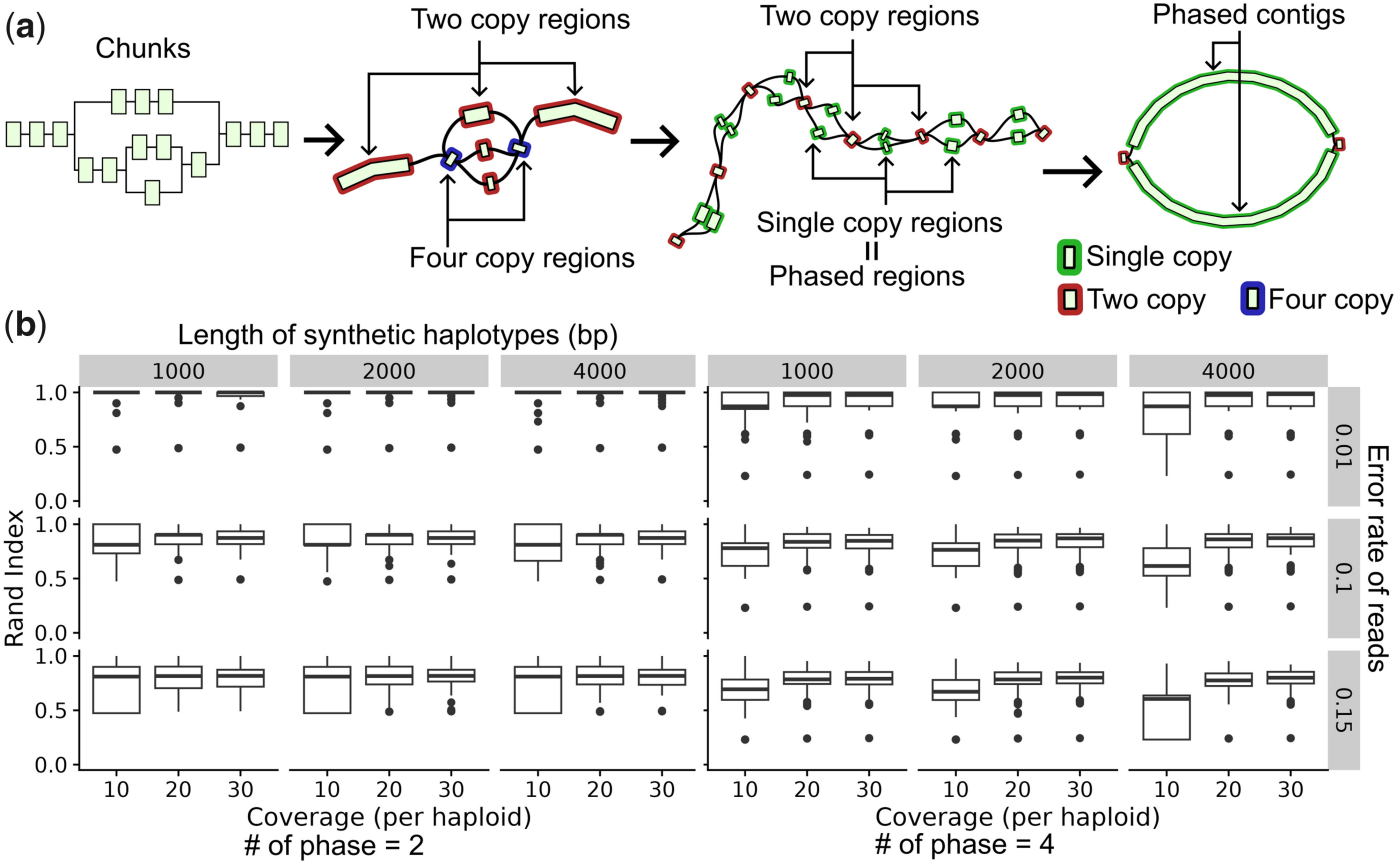
(a) Schematic illustration of how JTK produce the diploid assembly. JTK checks the connection between chunks and then constructs an intermediate assembly graph (the first graph to the second). Then, JTK separates the chunks into their copies to generate a partially phased assembly (the second to the third). Finally, it unzips the two copy regions to get the final assembly. We used Gfaviz (Gonnella *et al.* 2019) for visualization. (b) The result of the clustering algorithm. The number of copies was two (the left) and four (the right). The rand index is the number of pairs that are in the same cluster or in different clusters in the correct and predicted clusterings divided by the total number of pairs.

We can approximate these numbers by dividing the number of alignments between the reads and the chunks by the genome-wide haploid coverage. For example, if the haploid coverage is 20 and there are 80 alignments between a chunk and the reads, we estimate the copy number of it as four (=80/20). To make this approximation accurate, we consider the copy numbers of neighboring chunks. For example, as shown in the second graph in Fig. 2a, if a chunk is adjacent to two chunks with copy numbers equal to two, we impose a constraint that the copy number of that chunk should be four. We devise an optimization problem to consider this constraint and the coverage (Section 5 in Supplementary Data).

To determine the length of the chunk, *L*, we consider the copy numbers and the coverages of the chunks. Specifically, longer chunks would be less repetitive because the chance to include the unique region increases. Since decomposing a chunk into its copies becomes more challenging as the copy number grows, longer chunks are preferable. In contrast, shorter chunks have more reads to be aligned. Because we will use variants to separate a chunk into its copies, and these variants are easy to detect if the coverage is high, it is better to have shorter chunks. After testing several values, we found that L=2000 balances the above tradeoff.

In summary, we have the chunks, the alignments between these chunks and the reads, and the copy numbers of these chunks. In the next section, we separate the chunk into multiple copies by using the reads and the alignments to produce a diploid assembly.

### 2.3 Separating the chunks into copies

Given a chunk with a copy number equal to *K*, we separate the chunk into *K* copies based on the variants found on the chunk. For example, suppose the chunk is in an SD or, equivalently, in a loop in the assembly graph (the four copy regions in Fig. 2a). We need to separate the chunk into four copies, each corresponding to a copy in the target region. In this section, we describe how to find the variants by using the reads aligned to the chunk and how to separate the reads on the chunk into the *K* clusters based on the variants in the reads.

To find variants on a chunk, we exploit the idea of pair-hidden Markov models proposed in the HGAP (Chin *et al.* 2013, Edge and Bansal 2019), which models the error pattern of a sequencer. Shortly, it can compute the probability of generating a read from a chunk, which we call the “likelihood” of the read.

Specifically, suppose there are *N* reads, $R=\{r_{1},\ldots ,r_{N}\}$, on a chunk *c*. We propose a two-step approach to call variants and separate the reads *R* into *K* clusters, $R_{1},\ldots ,R_{K}$.

First, to exhaustively search SNVs on a chunk, JTK introduces each possible SNV to the chunk and records how the log-likelihood changes for each read *r*. Here, we specify an SNV by its edit operation *e* (substitutions, insertions, or deletion) and its position *i* on the chunk. Then, we can denote the table of these changes as *P[r][i][e]*, which we call a “perturbation matrix”. We can compute the table in $O(NL^{2})$ time, where *L* is the maximum length of the reads (Chin *et al.* 2013, Section 6 in Supplementary Data). For example, $P[r_{1}][100][\text{del}]$ is how much the log-likelihood of the first read changes by deleting the 100th base of the chunk, and $r_{1}$ supports this deletion if it is positive. Thus, if P[r][100][del]>0 holds for around half of the reads, this deletion is likely to be an actual variant between the copies.

Precisely, for each possible SNV (i,e), we sum up the positive value of the perturbation matrix over the reads, $\sum_{r\in R}\max(P[r][i][e],0)$. This value gives how much log-likelihood we can improve by introducing the SNV on the chunk, and we collect SNVs giving a large value of the sum. We denote these collected SNVs as U={(i,e)}.

Note that these SNVs can contain false positives, which lowers the accuracy of the following clustering. To remove these unreliable variants, we discard variants that are in long homopolymer run, have strand biases, or small gain of the log-likelihood (Section 7 in Supplementary Data).

Second, to partition the reads *R* into the *K* clusters, we maximize the following objective function with respect to the clustering of the read, $R_{1},\ldots ,R_{K}$, such that the union is *R*, and $R_{k}$ and $R_{k^{\prime }}$ are disjoint for all $k\neq k^{\prime }$.


(1) $$\sum_{k=1,\ldots ,K}\sum_{(i,e)\in U}\max(\sum_{r\in R_{k}}P[r][i][e],0)$$

Shortly, $\sum_{r\in R_{k}}P[r][i][e]$ is how much log-likelihood would increase in the reads in $R_{k}$ by editing the *i*-th base of the chunk by *e*. To ignore the case where this value is negative and the cluster does not support the SNV, we put max(_,0) in (1). We use a sampling algorithm to find a nearly optimal solution (Algorithm 1).

Algorithm 1. Clustering of reads

**Input:** A perturbation matrix *P*, the variants *U*, and the copy number *K*

**Output:**$R_{1},\ldots ,R_{K}$ (a clustering of $\left\{r_{1},\ldots ,r_{N}\right\}$)

1: $R_{1},\ldots ,R_{K}\leftarrow$ randomly partition $r_{1},\ldots ,r_{N}$ into *K* clusters.

2: p← compute the objective function (1) ▹ Greater is better.

3: **for** *T* times **:**▹*T*=2000×n by default

4:   Select *i* and *j* randomly and move a random element in $R_{i}$ to $R_{j}$

5:   $\hat{p}\leftarrow$ compute the objective function (1)

6:   **if**$p<\hat{p}$**:**

7:    $p\leftarrow \hat{p}$

8:   **else**

9:    Set *p* to $\hat{p}$ with a probability $\text{exp}(\hat{p}-p)$; otherwise, restore the latest change made at Steps 4

10: **return**$R_{1},\ldots ,R_{K}$

Because of the high error rate in the ONT reads, the clustering (Algorithm 1) may fail to assign a read to the correct cluster. To fix this issue, JTK further improves the clustering on a chunk by integrating the results of nearby chunks (Section 8 in Supplementary Data).

In summary, to separate the chunks into their copies, we assign the reads on each chunk into its copies based on variants found by the perturbation matrix. The remaining tasks are determining the order of the separated copies and resolving regions without variants.

### 2.4 Serializing the separated copies

After the clustering, we infer how the copies of the chunks order in the target region by a graph where the nodes are the copies of the chunks, and an edge links two nodes adjacent in a read (Section 9 in Supplementary Data).

This graph is not necessarily fully phased into the two haplotypes at this stage because we cannot separate chunks without any variants, i.e. chunks corresponding to completely homozygous regions (Fig. 2a the third graph). We solve these homozygous regions by finding the correct path of each haplotype based on the co-occurrence of the nodes in the reads.

To generate the final assembly, we first concatenate chunks inside the path in the graph to obtain the draft sequences and then take consensus among the reads by using the perturbation matrix on the draft sequences. Specifically, we compute the perturbation matrix on the draft sequence *s* and take the sum over the reads to get $P_{s}[i][e]=\sum_{r}P[r][i][e]$. While there is a position and an edit distance (i,e) such that $P_{s}[i][e]$ is positive, we apply it to the draft sequence to get a better consensus.

After taking consensus, the nodes of the phased assembly are labeled either haplotype-phased or multicopy (Fig. 2a rightmost graph). We use the haplotype-phased contigs in the downstream analysis.

### 2.5 Compared pipeline and software

Currently, HiFi-assemblers are the first choice to assemble genomes in diploid resolution. Thus, we used assemblies from Verkko and HiFiASM with only HiFi reads with around 60-fold coverage, which is as expensive as 60-fold ONT reads.

Also, we ran three different approaches for ONT reads. The first approach is variant calling followed by phasing with the latest software. We used DeepVarinat (Poplin *et al.* 2018) and CuteSV (Jiang *et al.* 2020) to call variants, Longphase (Lin *et al.* 2022) to phase them, and Flye (Kolmogorov *et al.* 2019) to assemble the reads from the haplotypes separately. Second and third, we used PhaseBook (Luo *et al.* 2021) and PECAT (Nie *et al.* 2022).

To evaluate these programs, we generated ground truth haplotypes and simulated long-reads by NanoSim (Yang *et al.* 2017).

## 3 Result

### 3.1 Accuracy of clustering algorithm

We benchmarked the clustering algorithm (Algorithm 1) on simulated datasets. Specifically, we generated chunks of *L* bp in size (*L* = 1K, 2K, 4K) and produced copies of chunks to be phased that differed in one edit operation. Then, we simulated noisy reads with varying error rates and coverages from these synthetic copies. Finally, we carried out clustering these reads and measured the accuracy by the Rand index (Rand 1971).

The Rand index was around one minus the error rate when the copy number was two, and it was more than 0.8 when the copy number was four (Fig. 2b). This result showed the robustness of our method to errors and coverages, suggesting that clustering of noisy reads is possible with only one variant.

### 3.2 Comparison of assemblies on simulated reads

We assessed the performance of JTK by using simulated long reads.

First, we benchmarked on simulated haplotypes. Specifically, to simulate large SVs and sporadic SNVs between haplotypes, we synthesized a 1-Mb random sequence and introduced one 50-kb insertion, one 50-kb deletion, and 0.1% divergence to create synthetic haplotypes. From 60-fold ONT reads simulated from these haplotypes, JTK assembled 95% of the original genome in two contigs without errors. The remaining 5% corresponded to both ends of the regions, which are illustrated by the two short contigs in the rightmost graph in Fig. 2a.

To further validate our approach, we extract the COX and PGF haplotypes of the MHC region from the human genome build 38 as in the previous study (Luo *et al.* 2021) and we simulated 60-fold long reads.

Out of four tested software programs, JTK and PECAT output two haplotype-phased contigs, and the QVs are 62.0 and 59.1, respectively (Table 1).

In contrast, the combination of LongPhase and Flye failed to output haplotype-resolved contigs; namely, although LongPhase phased the reads throughout the entire region, Flye produced eight contigs on these phased reads. Also, the base-level QV of the assembly was around 20, presumably due to switching errors in LongPhase. Phasebook produced 423 highly fragmented contigs, and the N50 was around 230 kb. Note that diploid assembly in this region is impossible only by HiFi reads because they are usually shorter than 30 kb, and the longest homozygous region without any variants is 64 kb in this region.

Although JTKs performance comes at the expense of speed, JTK accurately assembles diploid sequences with noisy ONT long reads.

### 3.3 Maximum read length and the number of contigs

Read length is one of the key parameters that determine the quality of a genome assembly. Since the maximum length of the phased block is limited by the maximum length of the reads, we evaluated the relationship between the performance of JTK and the maximum length of the reads.

The reference genome we used was the synthetic and COX and PGF haplotypes from the previous section. By NanoSIM, we generated 60-fold datasets with 10 replicates for each parameter, changing the maximum length of the reads from 10 000 to 110 000 bp. We assembled these datasets with JTK and examined how the maximum length of the reads influences the number of phased contigs, which ideally should be two (=one per haplotype) (Supplementary Fig. S1).

On the synthetic datasets, the maximum length had to be above 40 000 bp for the number of contigs to be two. In contrast, on the COX and PGF haplotypes, JTK needed a length of more than 90 000 bp to achieve the optimal assembly.

Although more sophisticated software might be able to produce phased assembly of actual genomes with shorter reads, JTK needs reads of more than 90 000 bp for reliable results.

### 3.4 Assemblies on real reads

#### 3.4.1 Target regions and datasets

We measured the performance of JTK on eight real datasets with 60-fold coverages (Table 2 and Supplementary Figs S2–S5). These datasets were generated on the PromethION ultralong protocol sequencers (Section 1 in Supplementary Data). The accession numbers of the HG002 datasets used were from SRR18363756 to SRR18363760, and that of B080 was JGAS000580.

**Table 2.** Description of the dataset.

Sample	Region	Total (Mb)	N50 (kb)	Mean (kb)	Max (kb)
HG002	MHC	320	45	17	230
HG002	LILR + KIR	70	46	18	150
HG002	Chr1sub	320	42	18	211
HG002	SMN	240	45	18	325
B080	MHC	320	57	26	720
B080	LILR + KIR	70	64	28	533
B080	Chr1sub	320	59	26	830
B080	SMN	240	57	25	902

MHC region is chr6:28,381,458–33,301,940, LILR + KIR is the chr19:57M–58M, Chr1 sub is chr1:10M–15M and SMN is chr5:69,399,944–72,682,017 in the T2T-CHM13 reference version 2.0. Section 1 in Supplementary Data describes the sequencing protocol used to sequence B080.

**Table 3.** Comparison of the assemblies on six real datasets.

Region	Software	No. of contigs	ASM. size (kb)	QV
Chr1	JTK	2	10,177	27.5
HG002	LongPhase	6	10,324	24.7
	PECAT	14	10,753	16.8
	Verkko	133	10,845	35.3
LILR-KIR	JTK	2	2,175	25.4
HG002	LongPhase	4	2,484	21.2
	PECAT	5	3,047	12.5
	Verkko	29	2,416	35.0
MHC	JTK	2	10,163	27.7
HG002	LongPhase	3	10,542	22.9
	PECAT	7	10,387	20.1
	Verkko	113	12,044	36.8
Chr1	JTK	2	10,242	29.1
B080	LongPhase	5	11,116	28.5
	PECAT	4	9,603	21.7
	HiFiASM	118	12,284	40.5
LILR-KIR	JTK	2	2,976	27.5
B080	LongPhase	3	2,338	18.4
	PECAT	2	2,302	26.2
	HiFiASM	18	2,968	37.7
MHC	JTK	2	10,111	30.8
B080	LongPhase	3	9,502	19.3
	PECAT	3	10,205	27.6
	HiFiASM	59	15,874	55.6

The difference is the sum of the NM tag in minimap2 after alignment to baseline assemblies. Verkko means Verkko without Hi-C and ONT reads. HiFiASM means HiFiASM without Hi-C.

In the following sections, we refer to the LRC as leukocyte Ig-like receptor and killer-cell Ig-like receptor (LILR-KIR) region, as the LRC complex consists of LILR and KIR domains.

In addition to the highly diverged MHC and LILR-KIR regions, we included the 10- to 15-Mb region of chromosome 1, as this region contains a large divergence in a population (Porubsky *et al.* 2022). To demonstrate the limitations of JTK and other existing genome assemblers, we used spinal muscular atrophy locus, which we refer to as the SMN region (Vollger *et al.* 2022).

To compute the QV, We obtained the genome-scale diploid assembly by Verkko (Rautiainen *et al.* 2022) from the HG002 data with 100x HiFi, 85x ONT UL (>100 kb), and Hi-C dataset. For an in-house sequence dataset from a Japanese sample, which is referred to as B080 hereafter, HiFiASM (Cheng *et al.* 2021) with 60-fold HiFi reads with Hi-C was used to generate the assembly.

#### 3.4.2 General comparison of assemblies

JTK fully resolved the targeted regions, MHC, LILR + KIR, and 10–15 Mb in chromosome 1 from 60-fold ONT reads (Table 3). They showed high concordance (QV∼30) with baseline assemblies, which were assembled by HiFiASM or Verkko from high-coverage HiFi reads and ONT ultralong reads or Hi-C reads. In the JTK assemblies, around 95% of errors were due to incorrect estimation of homopolymer length. For example, out of 17 194 errors in the assembly on the MHC region of the HG002, only 790 were mismatches.

To further assess the quality of JTKs contigs, we called the variants by using JTKs contigs and baseline assemblies on hg38. We used dipcall (Li *et al.* 2018) version 0.3 and happy (Krusche *et al.* 2019) on the GIAB variant benchmark as in the previous study (Jarvis *et al.* 2022) (Table 4). Notably, the recall and precision of the JTK contigs for the SNPs were compatible with those of the baseline assemblies. For example, the recall of the JTK contigs on the 10- to 15-Mb subregion in chromosome 1, LILR-KIR, and MHC regions were 98%, 84%, and 19%, respectively, and those of the baseline assemblies were 99%, 85%, and 20%, respectively. Meanwhile, these metrics of the JTK contigs on the indel variants were much worse than those of the baseline assemblies. Similar to the analysis on the QVs, these results suggest that most of the consensus errors were short insertions and deletions.

**Table 4.** Performance of JTKs contigs and the baseline assemblies of HG002 against GIAB benchmark.

Region	Type	Software	Recall	Precision
Chr1	SNP	Baseline	0.99	0.85
		JTK	0.98	0.76
Chr1	Indel	Baseline	0.97	0.56
		JTK	0.58	0.07
LILR-KIR	SNP	Baseline	0.85	0.56
		JTK	0.84	0.52
LILR-KIR	Indel	Baseline	0.92	0.41
		JTK	0.57	0.07
MHC	SNP	Baseline	0.20	0.99
		JTK	0.19	0.95
MHC	Indel	Baseline	0.18	0.95
		JTK	0.13	0.19

We used the hg38 reference, dipcall, and hap.py to compute these metrics.

Although Verkko and HiFiASM output complete assemblies for the five datasets with HiFi and ONT or Hi-C, they produced 18–133 fragmented contigs without ONT or Hi-C depending on the size of the assembled regions and the coverage of the reads. In addition, the quality values of the HiFi-only assemblies might be overestimated due to the following reason. The baseline assemblies used to compute QV were constructed from the same HiFi reads used for the HiFi-only assemblies. Thus, remaining errors in baseline assemblies might be shared with HiFi-only assemblies and do not affect the QVs of HiFi-only assemblies.

The phasing pipeline, LongPhase with Flye, produced unsolved assemblies for all datasets except LILR-KIR in the B080 sample. For example, in the chr1:10- to 15-Mb region in HG002, the contigs were split at the SD-rich region (Supplementary Fig. S6). The QVs of the contigs varied among the dataset, but they ranged from 20 to 30 in general. One explanation for the low QV is switching errors inside the contigs. In other words, like in the case of the synthetic dataset (Table 1), LongPhase incorrectly phased the variants and made chimeric haplotypes. Another explanation is that Flye’s consensus module is not as accurate as JTKs, as Flye is mainly for fast chromosome-scale assembly while JTK is a megabase-scale assembler and tries to make the consensus as accurate as possible.

**Table 1.** Results of the assembly on the reads simulated from the composition of the COX and PGF haplotypes.

Software	No. contigs	Total bases (kb)	QV	Time (min)
JTK	2	9,502	62.0	40.6
PECAT	2	9,736	59.1	9.6
LongPhase + Flye	8	9,816	20.6	29.0
PhaseBook	423	75,357,275	–	31.5

PECAT, an ONT-only assembler, output fragmented assemblies. For example, the LILR-KIR region in the HG002 was decomposed into five fragments of the partially phased contigs (Supplementary Fig. S8). Similar to the phasing pipeline, the contigs terminated near repetitive sequences and overestimated the segmental duplications in the KIR region. In addition to this structural misassembly described, we noticed that PECAT produced redundant contigs in the LILR-KIR assembly (Supplementary Fig. S8).

In contrast to these regions, on the SMN region, all tested software (JTK, LongPhase pipeline, PECAT, and HiFi-only assembly) failed to assemble both HG002 and B080 datasets into phased assemblies. However, the number of contigs produced by JTK was smaller than that produced by the other software (Supplementary Figs S15 and S16). In particular, on the B080 dataset, JTK assembled into four haploid contigs, leaving one repeat unresolved, while the other software produced more fragmented contigs.

#### 3.4.3 Analysis of the assemblies obtained by JTK

In the MHC region in HG002, we find a highly diverged region at the 1 Mb of the assembled contigs upstream of the class II genes (Supplementary Fig. S9). In this expanded region, these two haplotypes contained four genes annotated by Liftoff (Shumate and Salzberg 2021). Specifically, the four shared genes are three HLA class II genes (*HLA-DRB1*, *HLA-DRB6*, *HLA-DRB5*) and *RNU1-61P*. By aligning the MHC contigs from HG002 and B080, we searched the corresponding regions in B080 (Supplementary Fig. S10). These regions showed low sequence similarity, and we found that the two haplotypes in B080 had the same set of genes annotated by Liftoff. These results suggest that the haplotypes within this region share the same set of genes even when the sequence conservation is reduced.

**Figure 3.**
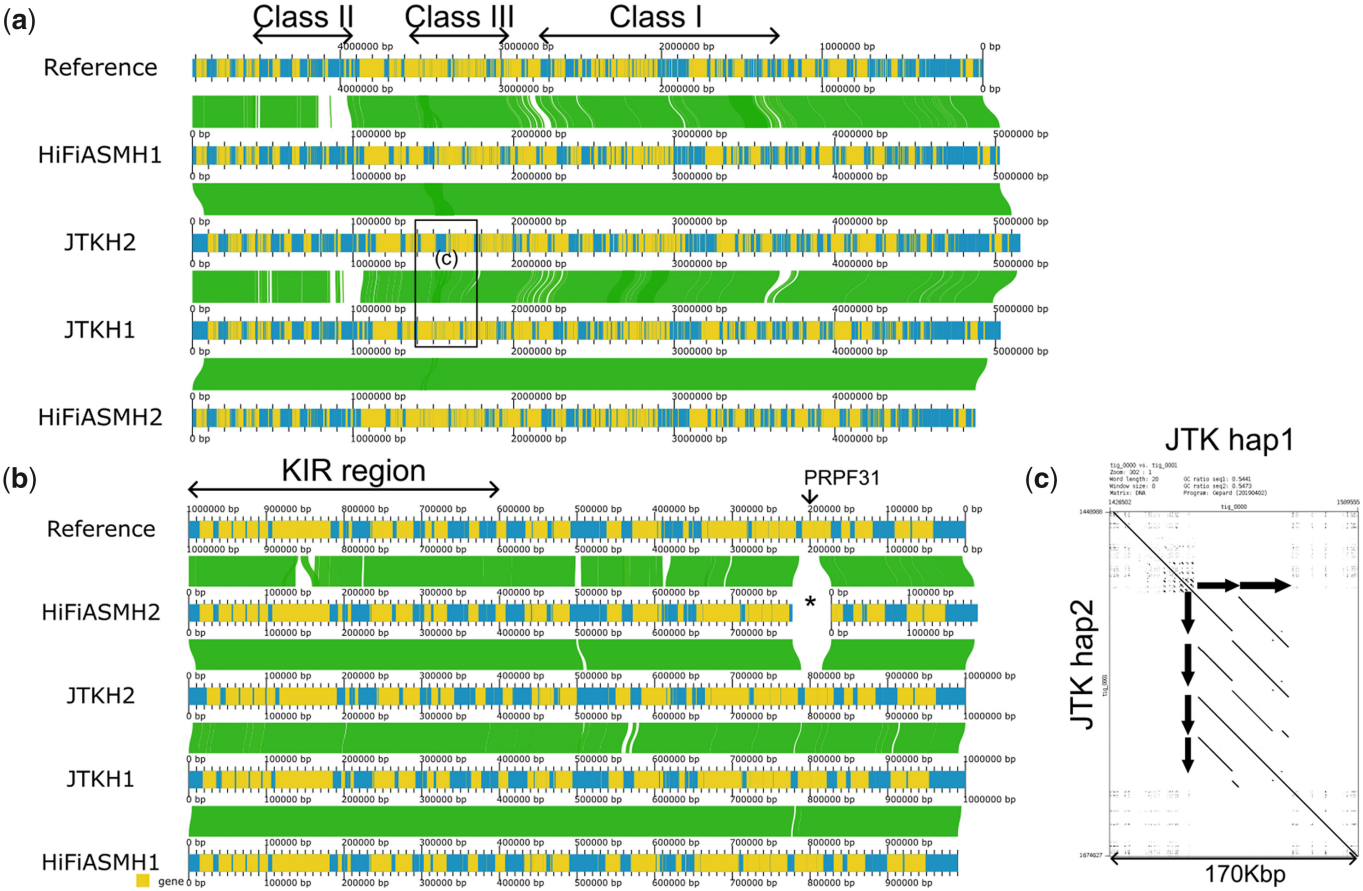
Comparison between the assemblies in the real datasets by Gepard (Krumsiek *et al.* 2007) and AliTV (Ankenbrand *et al.* 2017). Blue bars, yellow bands, and green ribbons represent contigs, gene annotations, and alignments, respectively. (a) The MHC region in B080. A large segmental duplication (the black box) is enlarged as (c). (b) The LILR-KIR in B080. The * mark indicates the region missing from the HiFiASMs contig. (c) An enlarged dotplot on the MHC region in B080. The arrows indicate the copies of the segmental duplications.

In the MHC class III region in B080, we uncovered a copy number variation of 50-kb segmental duplication (two copies in one haplotype and four in the other, Fig. 3c), which we validated by a HiFi+Hi-C assembly. Both haplotypes had the same two annotated genes, *C4A* and *C4B*, and six annotated pseudogenes. These genes have different copies from individual to individual (https://www.genecards.org/cgi-bin/carddisp.pl?gene=C4B). By examining the regions in the HG002 contigs corresponding to these class III regions, we found that two haplotypes in the HG002 sample had two copies of the 50-kb segmental duplications and the same annotated genes (Supplementary Fig. S11). These results demonstrated the ability of JTK to detect the copy number variations of them.

In the LILR-KIR region in B080, we confirmed JTKs assembly by observing that there were no coverage drops and by running JTK with a different seed for pseudorandom number generators to obtain the same structure. In contrast, HiFiASMs assembly contained a 26-kb gap around 57.2 Mb in chr19 of the T2T assembly. This gap contains two genes, *PRPF31* and *AC245052.4* (3b), which were annotated in the JTKs contigs. We found *KIR3DL3, KIR2DL3, KIR2DP1, KIR2DL1, KIR2DL4, KIR3DL1, KIR2DS4*, and *KIR2DL2* in both haplotypes in this order from the centromere to the telomere direction, suggesting both haplotypes were haplotype A, i.e. the major haplotype of this region. There were as many as 0.2% SNVs between two haplotypes (2574 substitutions out of 1.2 Mb alignment) and we discovered a large (∼3 kb) expansion of CT-rich sequence between the haplotypes, which was confirmed by alignment by the ONT reads (Supplementary Fig. S14). In summary, the complete diploid assembly enables us to find significant divergences between the major haplotype in the KIR-LILR region.

## 4 Discussion

### 4.1 Comparison between JTK and other assemblers

For years, diploid assembly of erroneous reads seemed difficult because naive sequence identity between two reads would mix up SNVs between haplotypes and sequencing errors. JTK overcomes this issue and assembles genomic regions in diploid resolution from long-noisy reads.

The contigs produced by JTK were more contiguous than those from the HiFi-only assembly from Verkko or HiFiASM. As the distance between adjacent variants can exceed 30 kb, it is impossible to phase these “SNV-deserts” by HiFi-reads. Therefore, the accuracy of the reads is not a sufficient condition for the diploid assembly, while the length of the reads can be.

Compared to phasing tools such as LongPhase, JTK can overcome errors caused by complex SVs and SDs. For example, the phasing-based method caused switching errors in the synthetic reads from the COX and PGF haplotypes, which were highly heterozygous. In contrast, JTK assembled these haplotypes correctly. The critical point might be using the chunks instead of the single reference sequence. If the heterozygosity is high, JTK can use different chunks to represent the region, which avoids the reference bias in the variant callers and the phasing tools.

PECAT is also an ONT-based assembler. However, in terms of contiguity and base accuracy, JTK generated better contigs. For example, in the 10- to 15-Mb region in chromosome 1, while PECAT produced 14 contigs with QV = 16, JTK output two contigs with QV >27, which fully resolved this region. One explanation is that while PECAT first corrects errors and then assembles the contigs, JTK uses uncorrected reads and variants on chunks. As error correction can result in overcorrection, PECAT can overlook essential SNVs and produce shorter phased blocks.

### 4.2 Limitation of JTK on genome-scale assembly

We have compared JTK with other software on three different datasets including one synthetic dataset and two real human datasets. Here, we discuss the limitations of JTK and whether it can assemble the entire genomes.

First, JTK cannot currently produce phased assemblies on at least two types of regions, and thus cannot be applied to the entire genome. One situation is when the target region is a long stretch of tandem repeats (TRs), such as centromeres. If a chunk is sampled from a TR, there would be too many alignments between the chunk and the reads for JTK to process in a reasonable time.

Another situation is the region containing multicopy (>10) long (>10 000 bp) exact repeats. These repeats complicate the assembly graph and lead to suboptimal assembly. One such region is SMN, and all ONT-based assemblers tested in this study failed to assemble this region perfectly. Since the SMN region is considered one of the most difficult regions to assemble (Vollger *et al.* 2022), we need better datasets and algorithms to obtain phased contigs reliably.

As future work, we propose two approaches to solve these problems. To deal with long TRs, one approach is to separate reads from these TRs beforehand and assemble these reads by estimating the length and variants of the repeats. For example, DeepRepeat (Fang *et al.* 2022) is a tool to find TRs from the ONT reads. To deal with the highly repetitive exact repeats such as SMN, one approach is to use a different formalization at the graph construction and simplification steps. For example, by considering each chunk as a character, we can use string graph-based assembly, where the alignments are based on the chunks. This approach would simultaneously take into account the variations between chunks and may have more power than the current implementation.

### 4.3 The assemblies in the real datasets

When comparing two haplotype-resolved contigs, their differences must not be artifacts of the assembler. Speaking of the MHC region in both samples, we think the differences we observed were actual ones because the class II and III regions are highly variable, and complementary datasets (HiFi+Hi-C) produced the same structure (Jensen *et al.* 2017).

In the HG002 sample, we picked the upstream region of the MHC class II region, and in the B080 sample, we focused on one segmental duplication in the MHC class III region. In both cases, the gene prediction suggested that the gene contents were the same between the two haplotypes, only varying the noncoding regions or the copy number of *C4* genes. We hypothesize from these observations that the haplotype differences around the MHC class II/III region have a small effect on the gene repertoire. In other words, the variations in the MHC region would not remove genes or insert a new gene but introduce SNVs or copy number variations.

Regarding the LILR-KIR region in the B080, there may be a switching error at the position where HiFiASM breaks contigs. Nonetheless, we support the correctness of these haplotypes because the gene predictions were consistent with the reference, and JTK assembled nearly identical haplotypes with different seeds for pseudorandom number generators.

On these assemblies, the annotation suggested that both haplotypes are the major haplotype in the Japanese population (Morishima *et al.* 2010). Nonetheless, although there would be a small number of consensus errors remaining, we saw around 0.2% substitution between haplotypes, confirming the high divergence among the major haplotype.

Because datasets with sufficient Nanopore ultralong reads from individual humans are very limited, our approach was validated using two datasets and one synthetic dataset, but should be tested against population-scale datasets in the future. Indeed, projects are already underway to obtain ultralong leads for ONT on a number of samples. In the future, the diploid assembly would be widely available to describe the haplotype structure in fine detail, paving the way for medical applications of personal genomics.

## 5 Conclusion

Diploid assembly is an essential component to elucidate human genetic diversity. However, existing approaches either fail to assemble complex regions or require extensive data from different technologies. The presented software, JTK, assembled both simulated and real reads from a single long-read technology on regions previously thought to be hard to assemble. Combined with phasing tools for regions with low heterozygosity, regional diploid genome assemblers like JTK would be a cost-effective selection in the coming era of comparative pan-genomics.

## Supplementary Material

btad398_Supplementary_Data
