# Supplementary material for: JTK: targeted diploid genome assembler
Source: Bioinformatics. 2023 Jun 24;39(7):btad398. doi: 10.1093/bioinformatics/btad398 (PMC10320103; doi:10.1093/bioinformatics/btad398)
Supplement: btad398_Supplementary_Data [file btad398_supplementary_data.zip › jtk_suppl_text.pdf]

Supplementary documents for JTK

Bansho Masutani, Yoshihiko Suzuki, Yuta Suzuki,
and Shinichi Morishita

April 1, 2023

1 Cell culture, DNA isolation, and library preparation for Nanopore DNA sequencing

For Nanopore Sequencing, the B-lymphoblast cell line was obtained from the Japanese Collection of Research Bioresources (Japanese B cell DNA bank), National Institutes of Biomedical Innovation, Health and Nutrition in Japan. Cells were cultured at UNITECH Co., Ltd (Chiba, Japan).

Briefly, cryopreserved PSCCA0080 cells were thawed and cultured in a growth medium (RPMI-1640 medium with 10% FBS) at 37℃ in the incubator supplied with 5% of CO2 for 3-4 days. For passage, 5-8 fold dilution of cell suspension was seeded into a T75 flask or T150 flask and incubated at 37℃, 5% CO2 for 3 days. PSCCA0080 cells were suspended at six million cells/ml in RPMI-1640 medium with 20% FBS and 6% dimethylsulfoxide (DMSO) and frozen.

According to the ULK_9124_v110_revF_24Mar2021 version of Nanopore Ultra-Long DNA Sequencing Kit (SQK-ULK001) protocol, input DNA was extracted using NEB Monarch HMW DNA Extraction Kit for Tissue and ultra-long DNA libraries were prepared. The input DNA was extracted from 6 million cells per ultra-long DNA library. The eluted ultra-long DNA quality was measured using the dsDNA BR Assay Kit with a Qubit fluorometer (Thermo Fisher Scientific) and the Pippin Pulse system (Sage Science). Ultra-long DNA library preparation required high-quality, high-molecular-weight input DNA with a majority of the DNA fragments >150 kb as determined by the Pippin Pulse using a 0.75% agarose gel and the 5-kb–430-kb protocol.

Nanopore libraries were prepared using Nanopore Ultra-Long Sequencing Kit (SQK-ULK001, ONT) and carried out on a Nanopore PromethION sequencer. FLO-PRO002 R9.4.1 PromethION flowcells were used and the run length setting was 72 hours. The DNA library was viscous and did not readily flow through the Inlet port into the flow cell. We applied negative pressure in the flow cell as described in the protocol. To increase data output, a second library (at 24 hours after the run was started) and a third library (at 48 hours after the run was started) of ultra-long DNA were reloaded straight after flushing a flow cell using Nanopore Flow Cell Wash Kit (EXP-WSH004).

2 Selecting reads from the target regions

To filter reads from the target regions, we used the following pipeline:

1. Align all the ONT reads to the T2T-CHM13 version 2 by minimap2.
   `minimap2 -a --secondary=no -x map-ont $T2T $READS | samtools view -OBAM | samtools sort -OBAM`

2. Index the bam file by `samtools index`

3. Filter out the reads.
   `samtools view -OBAM $INDEXED_BAM $REGION | samtools fastq`

The accession number of the used HG002 datasets were from SRR18363756 to SRR18363760, and that of B080 was JGAS000580. The version of the `minimap2` was 2.24-r1122 ([5]), and `samtools` was 1.15.1.

For the B080 datasets, we used `guppy_basecaller` version 5.0.16+b9fcd7b with `-c dna_r9.4.1_450bps_hac_prom.cfg`.

3 Commands in the Benchmark software

3.1 Parameters for LongPhase and Flye

We used the following pipeline to use `LongPhase` Version 1.2 ([7]) and `Flye` version 2.9 ([3]) to assemble diploig contigs.

1. Map the reads to the T2T-CHM13 reference by `minimap2 --MD -a -x map-ont | samtools sort -OBAM`

2. Call variants by `DeepVariant` by `run_pepper_margin_deepvariant call_variant --ont_r9_guppy5_sup`.

3. Call SVs by `cuteSV --report_readid --genotype --max_cluster_bias_INS 100 --diff_ratio_merging_INS 0.3 --max_cluster_bias_DEL 100 --diff_ratio_merging_DEL 0.3`, which was the recommended parameters provided by the authors.

4. Phase these SNVs and SVs by `LongPhase` by `longphase_linux-x64 phase --ont`. Alignments were tagged by `longphase_linux-x64 haplotag -s`.

5. Assemble the reads assigned to each haplotype with unphased reads by `flye --nano-raw` with appropriate `--genome-size` option.

3.2 Parameters used in variant call analysis

To benchmark the accuracy based on the assemblies, we used the v4.2.1 GIAB benchmark variants with GA4GH ([9]), dipcall version 0.3 ([6]), and "hap.py" version 0.3.15 ([4]). We passed the parameter "-z200000,10000" to minimap2

and filtered the GAP2 variants as in the previous study ([2]). We parsed the output produced by "hap.py."

The GIAB variant benchmark uses the human reference genome build 38 as the reference genome, while we used the telomere-to-telomere assembly as the reference genome. Therefore, we had to convert the coordinates from T2T to hg38, and below is the table of converted ranges of MHC region, KIR region, and Chr1:10M-15Mbp. These values were calculated using the LiftOver service provided by the Broad Instutute (`https://liftover.broadinstitute.org/`).

Region name	interval on the T2T	interval on the hg38
MHC	chr6:28,381,458-33,301,940	chr6:28,510,029-33480577
LILR-KIR	chr19:57M-58M	chr19:53,920,293-54,905,697
Chr1:10M-15M		10,456,383-15,555,303

Table 1: Conversion between the intervals on the T2T and the intervals on the HG38.

4 Determine the chunks from ONT reads

The initial step of JTK is to determine a set of 2000 bp strings (*chunks*) to represent the underlying target region. Assuming the approximate length of the region is G bp, we randomly sampled 2000 bp substrings in $G/2000$ times from the reads as chunks. As a result, we have one-fold coverage by these chunks over the target region. Ideally, these chunks do not overlap and are uniformly distributed on the target region. However, the target region can have sub-regions without sampled chunks and make gaps in the assembly. We need to fill these gaps. Also, some chunks overlap, and these redundant chunks slow down the downstream analysis.

To fill the gaps in the target region, we map the chunks to the ONT reads, search substrings longer than 2000bp in reads where any chunks do not align, and sample new chunks from these substrings. We repeat this additional sampling until we can not get any additional chunks.

We overlap chunks with each other by `minimap2 --eqx -P` and iteratively remove chunks with the highest number of overlaps until we have no overlaps.

After these two follow-ups, the sampled chunks do not overlap, and there is no under-sampled region in the underlying region.

5 Copy number estimation of chunks

A chunk sampled in Section 4 can represent multiple similar sequences in the target region, such as homologous sequences between haplotypes or similar repeats, which we call *copies* of the chunk. To fully assemble the region, we

separate a chunk into each copy except homozygous regions without any variants. To this end, we first need to determine how many copies a chunk has in the target region, which we refer to as the *copy number* of the chunk.

Intuitively, to estimate copy numbers, we align the chunks to the reads, which tell two things. For one thing, they tell how many times a chunk is aligned to the reads. We can approximate the copy number by dividing it by the haploid coverage. For the other, the alignments tell the positions of the chunks aligned in the reads, which reveal how the copy numbers relate to each other. For example, if two chunks are always aligned side-by-side in the reads, these chunks must have the same copy number. We can fine-tune the approximated copy numbers from this information.

In the following section, we explain the algorithm to estimate the copy numbers. We first construct a graph called *chunk graph* from the chunks and the reads. Then, we convert the chunk graph into another graph called *chunk double-stranded graph* to derive an efficient algorithm for estimating copy numbers.

5.1 Construction of a chunk graph from the chunks, the reads, and the alignment between them.

Suppose two chunks are mapped to the same read, and there are no other chunks between them in the read. Since the read is a subsequence of the target region, these two chunks should be close to each other in the target region, and we call these two chunks *adjacent.* [1] The alignments between the chunks and the reads tell which pairs of chunks are adjacent. We can summarize these adjacencies among the reads by creating a graph where the nodes are the chunks, and we draw edges between adjacent chunks (Fig. S17).

Here, to make the graph represent the underlying target region, we need to consider two factors – the alignment direction and the strand direction.

First, a chunk is aligned either forward or reverse direction. For example, suppose three chunks u, v, and w are aligned to the same read in the forward direction (the first read in Fig. S17). We have two edges in this read; one connects the last base of u to the first base of v, and the other connects the last base of v and the first base of w. These bases are different, and we distinguish them by introducing H and T to represent a chunk's first (*head*) and last (*tail*) bases. For example, we represent the edge connecting the last base of u and the first base of v an edge from (u, T) to (v, H).

Second, although the reads have two directions (forward or reverse), we regard these directions as the same because they are essentially the same. For example, the first read in Fig. S17 has two chunks, u and v, aligned in the forward direction in this order. In contrast, the second read has two chunks, v and u, aligned as the reverse complement. These edges should be the same in the graph because they represent the same situation – they connect the last base of u and the first base of v. Thus, we treat an edge as undirected, i.e., we consider $(u, T) \leftrightarrow (v, H)$ to be the same as $(v, H) \leftrightarrow (u, T)$.

[1] Precisely, two copies of these chunks in the underlying target region are adjacent.

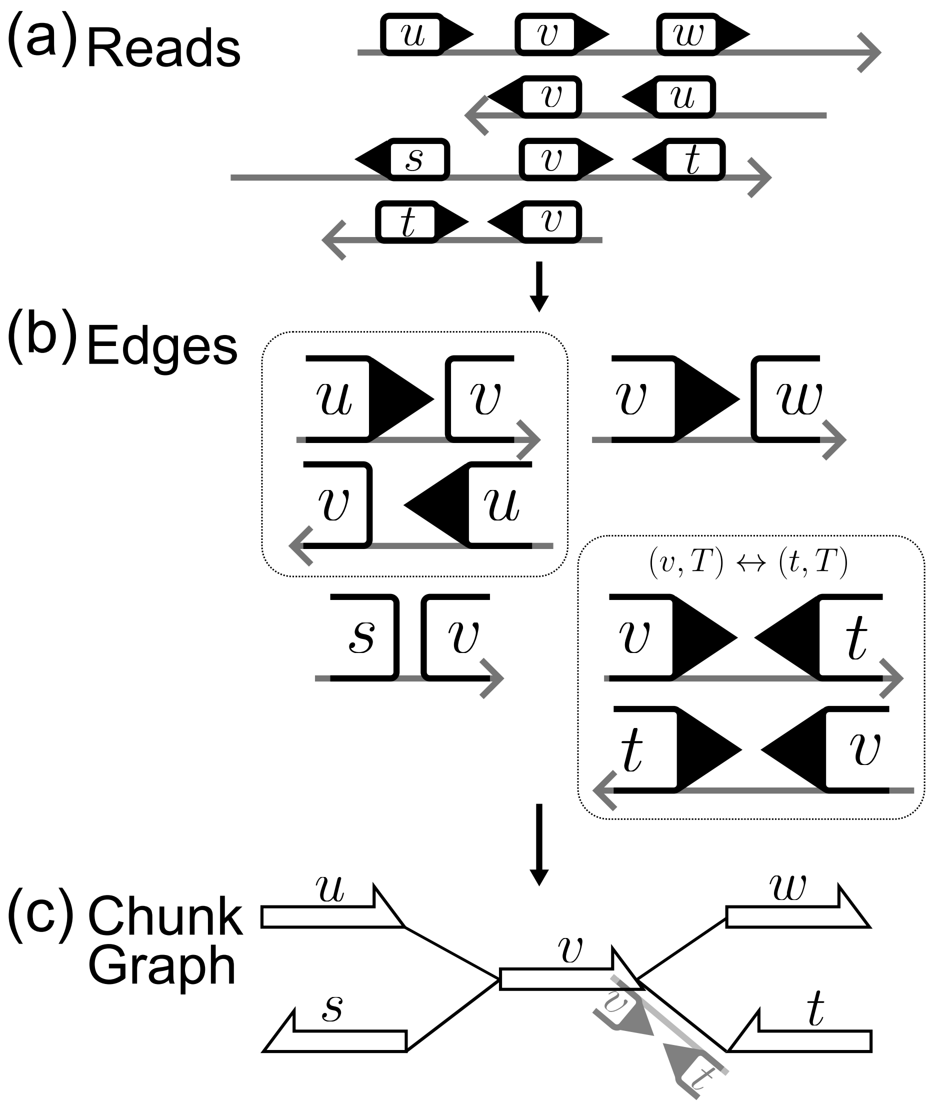

Figure S17: The conversion from the reads to the chunk graph. (a): A set of the reads (gray arrows indicate the sequence direction). The arrows at the side of the chunks indicate the alignment direction. (b): The set of edges from the reads. Each edge represents a connection of nodes and the direction of these nodes. We regard the edges in the dotted box as the same edge to erase the strand information of the reads; For example, the third and the fourth read have the same edge, $(v, T) \leftrightarrow (t, T)$. (c): The chunk graph converted from (b). We put the edge $(v, T) \leftrightarrow (t, T)$ in the graph as an example.

By considering these two factors, we represent the reads (Fig. S17a) as a *chunk graph* (Fig. S17c) $G = (V, E)$ as follows:

- The nodes V are the chunks.
- The edges $E \subseteq (V \times \{H, T\}) \times (V \times \{H, T\})$ contains $(v, p) \leftrightarrow (u, q)$ if and only if there is a read that connects the p (Head or Tail) position of the chunk v and the q (Head or Tail) position of u.
- Rigorously, we should define $V \times \{H, T\}$ as the nodes, but for simplicity, we refer to the set of chunks V as *nodes*.

A chunk graph has three types of coverages to approximate the copy numbers of the chunks. First, for each node v, we count the number of alignments between the reads and the chunk represented by v and call it the coverage of the node, c_v.

Second, for each edge e, we count the number of e in the reads, i.e., we count the occurrences of the adjacency, and call it the coverage of the edge, c_e.

Lastly, assuming the target region is in a diploid genome, we divide the average coverage of the chunks by two and denote it as the global haploid coverage, $\bar{c}$. Formally,

$$\bar{c} = \sum_{v \in V} c_v / 2|V| \tag{1}$$

Based on these coverages, we approximate the copy numbers of nodes as $c_v/\bar{c}$ and those of edges as $c_e/\bar{c}$. [2]

We improve these simple estimations by considering the structure of the chunk graph. For example, suppose the node v in Fig. S17 corresponds to a segmental duplication in the underlying diploid genome. Also, suppose that the nodes u, s, w, and t are flanking regions to the segmental duplication, and the copy numbers of these nodes are two. Here, the copy number of v should be four, which is the same as the sum of the copy numbers of u and s, or that of w and t.

In general, a node's copy number is equal to the sum of the copy numbers of the edges at either end (the dotted boxes in the chunk graph in Fig. S18). In the next section (Section 5.2), we present the optimization problem considering these consistency conditions and the coverages (c_v, c_e, and $\bar{c}$).

5.2 Converting a chunk graph to a double-stranded graph to estimate copy numbers

Formally speaking, we denote the copy number of a node v and an edge e as $\#_N(v) \in \mathbb{N}$ and $\#_E(e) \in \mathbb{N}$, respectively. Because these copy numbers should explain the observed coverages, the estimated copy numbers would minimize the sum of squared error (SSE):

[2] Precisely, we round them to the nearest integer.

$$\text{SSE} = \sum_{v\text{:nodes}} \|c_v - \#_N(v)\bar{c}\|^2 + \sum_{e\text{:edges}} \|c_e - \#_E(e)\bar{c}\|^2 \tag{2}$$

Also, as noted in the previous section, the consistency condition of the copy numbers below needs to be satisfied for each node v:

$$\#_N(v) = \sum_{e=(\_,\_)\leftrightarrow(v,H)} \#_E(e) \text{ if there is an edge to } (v,H) \tag{3}$$

$$\#_N(v) = \sum_{e=(v,T)\leftrightarrow(\_,\_)} \#_E(e) \text{ if there is an edge from } (v,T) \tag{4}$$

Combining the constraints on the graph, we have the following constrained minimization problem:

- Input: a chunk graph $G = (V, E)$ and coverages c_v, c_e, and $\bar{c}$.
- Output: Copy numbers on the nodes ($\#_N(v) \in \mathbb{N}$) and edges ($\#_E(e) \in \mathbb{N}$), which
  - minimize the sum of squared error (Eq. (2)).
  - satisfy the consistency conditions (Eq. (3), Eq. (4)) for each v.

How can we solve this optimization problem? One approach is to change $\#_N(v)$ and $\#_E(e)$ to reduce the SSE while maintaining consistency. As $\#_N(v) = 0$ and $\#_E(e) = 0$ for all nodes and edges satisfy the consistencies, we initialize the copy numbers as zero. The problem is how to change these copy numbers without breaking the consistencies.

Some paths and cycles in the chunk graph are helpful for this purpose. For example, think about the path in Fig. S18a consisting of u, $(u, T) \leftrightarrow (v, H)$, v, $(v, T) \leftrightarrow (t, T)$, and t. Incrementing all the copy numbers of nodes and edges in this path does not break the consistency conditions because whenever we increment the right side of condition (3) or (4), we increment the left side of these conditions and vice versa. We call these paths and cycles *balancing* because they "balance" the equation (3 and 4).

We summarize our approach so far as follows:

1. We initialize the copy numbers of edges and nodes to be zero.
2. We iteratively update the copy numbers along a balancing path or cycle to reduce the SSE (Eq. (2)).

Nonetheless, not all paths are balancing paths, and so are cycles. For example, u, $(u, T) \leftrightarrow (v, H)$, $(v, H) \leftrightarrow (s, H), s$ is a valid path in Fig. S18a, but we can not increment the copy numbers along this path without breaking the consistencies. How can we modify the structure of the chunk graph so that we can find balancing paths and cycles easily?

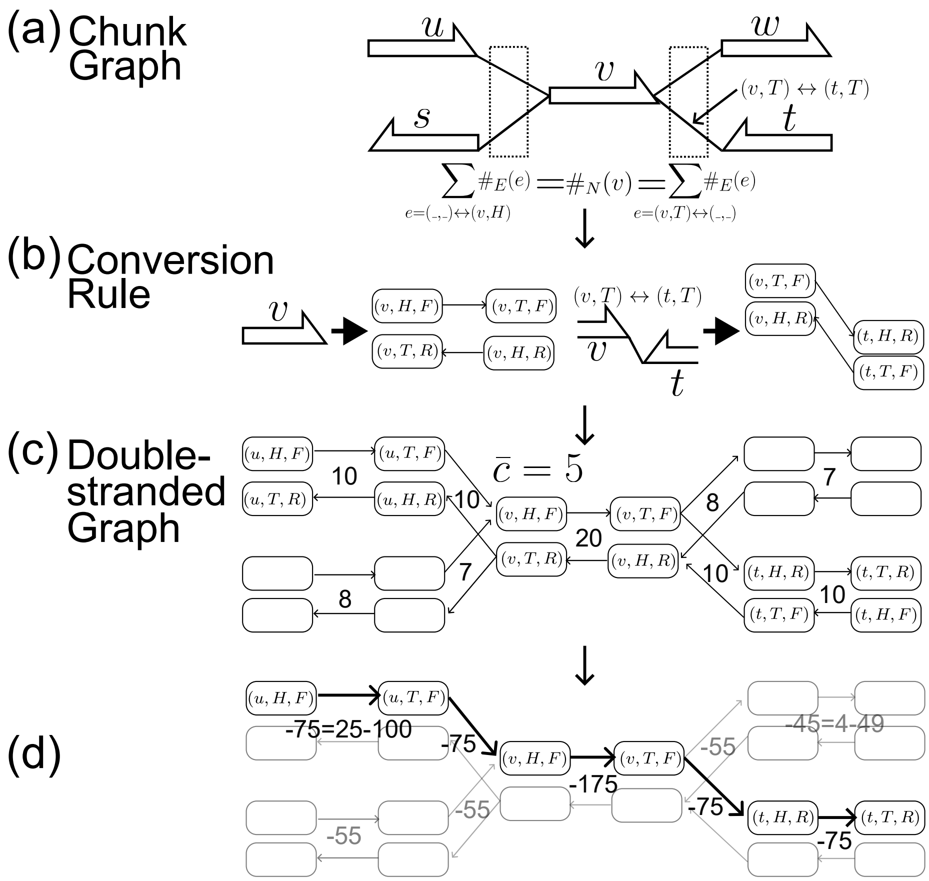

Figure S18: (a): The consistency condition on the chunk graph (Fig. S17c). Each head or tail position of the nodes has a consistency condition if there are edges connected to it. (b): The conversion rule for the double-stranded graph. We replace each node v with four nodes and two edges. We also replace each edge e with two edges. (c): The double-stranded graph converted from (a) by the conversion (b). The average coverage $\bar{c}$ is 5, and we label each edge by the coverages. (d): Initial iteration of the algorithm to estimate the copy numbers. We label each edge by how the sum of squared error decreases. For example, because we initialize all copy numbers as zero, the average coverage $\bar{c}$ is five, and the coverage of the edge $(u, H, F) \to (u, T, F)$ is ten, the weight of this edge is $\|10 - 1 \times \bar{c}\|^2 - \|10 - 0 \times \bar{c}\|^2 = -75$. The thick edges show the path selected in this iteration. (u, H, F) is the source and (t, T, R) is the sink of this path.

To this end, we introduce the strand information to the chunk and convert the chunk graph G into another directed graph as follows (Fig. S18b). Intuitively, after this conversion, every path should interchangeably traverse an edge and a node in the chunk graph.

1. Substitute v with four nodes representing the first/last base of the nodes and the forward/reverse strand. Formally, let H and T be the head (H) and the tail (T) of the nodes as before, and F and R be the forward strand (F) and the reverse strand (R). Then, we substitute v with $(v, H, F), (v, T, F), (v, H, R)$, and (v, T, R). For example, (v, H, F) is the head position in the forward strand of v.

2. For each v, we connect the head node to the tail node in both strands. Namely, we add two edges, $(v, H, F) \to (v, T, F)$ and $(v, H, R) \to (v, T, R)$. Hereafter, we will not add any edges from the heads, i.e., $(\_, H, F)$ or $(\_, H, R)$. Thus, whenever we reach $(\_, H, F)$ or $(\_, H, R)$, we need to go to the corresponding tail positions, i.e., $(\_, T, F)$ or $(\_, T, R)$. This property forces us to traverse an edge and a node interchangeably in the original chunk graph.

3. For each edge in the original graph, we split the edge into two directed edges representing the forward and reverse directions. For example, the edge $(v, T) \leftrightarrow (t, T)$ would be $(v, T, F) \to (t, H, R)$ and $(t, T, F) \to (v, H, R)$ (Fig. S18b). In general, we replace an edge $e = (v, p) \leftrightarrow (u, q)$ as follows. The first edge is a directed edge from v to u.

$$
\begin{aligned}
(v, T, F) &\to (u, H, F) \text{ if } p = T \text{ and } q = H \\
(v, T, F) &\to (u, H, R) \text{ if } p = T \text{ and } q = T \\
(v, T, R) &\to (u, H, F) \text{ if } p = H \text{ and } q = H \\
(v, T, R) &\to (u, H, R) \text{ if } p = H \text{ and } q = T
\end{aligned}
$$

   As the edge e is undirected, the second edge is a directed edge from u to v defined similarly.

$$
\begin{aligned}
(u, T, F) &\to (v, H, F) \text{ if } q = T \text{ and } p = H \\
(u, T, F) &\to (v, H, R) \text{ if } q = T \text{ and } p = T \\
(u, T, R) &\to (v, H, F) \text{ if } q = H \text{ and } p = H \\
(u, T, R) &\to (v, H, R) \text{ if } q = H \text{ and } p = T
\end{aligned}
$$

We call this graph the *double-stranded graph* because we introduce F and R to express the double-strandedness. We also refer to a node without any incoming edges as a *source* node and a node without any outgoing edge as a *sink* node.

The double-stranded graph has a useful property; all cycles and all paths from source nodes to sink nodes correspond to balancing cycles and paths in the original chunk graph. For example, the thick nodes and edges in Fig. S18c is a

path from the source node (u, H, F) to the sink node (t, T, R) corresponding to the balancing path consisting of u, $(u, T) \leftrightarrow (v, H)$, v, $(v, T) \leftrightarrow (t, T)$, and T in the original chunk graph. By definition of the balancing path, changing copy numbers along with this path does not break the consistency condition.

Thus, to optimize the SSE (Eq. (2)), we initialize the copy number of the nodes ($\#_N(v)$) and edges ($\#_E(e)$) as zero and change copy numbers along a cycle or a path in the double-stranded graph until we can not decrease the SSE (2). For example, suppose we set all the copy numbers to zero and then increment the copy numbers along the path depicted in Fig. S18d. The squared error of the edge $(u, H, F) \to (u, T, F)$ changes from $\|10 - 0 \times 5\|^2 = 100$ to $\|10 - 1 \times 5\|^2 = 25$, decreasing by 75. Summing up all the changes, the SSE decreases by 475. We continue this procedure until the SSE does not decrease.

Nonetheless, the above procedure is not rigorously defined in two points.

First, we need to determine how much the copy numbers we will change along a path or cycle in each iteration. In addition, we need to select a path or cycle from possibly many paths and cycles.

Shortly, for the first issue, we decide whether to increment or decrement the copy numbers at each iteration at random. As the copy numbers are integers, it is reasonable to assume that we can reach a nearly-optimal solution by incrementing or decrementing the copy numbers.

For the second issue, for each node and edge, we compute how the SSE (Eq. (2)) will change if we change the copy number of the node or edge. By summing up these changes along a path or cycle, we can also estimate how the SSE will change in total and thus prioritize the paths and the cycles.

Here, suppose we are going to increment the copy numbers. Then, for each edge in the double-stranded graph, we compute how the SSE changes when we increment the copy number of it. Because the increment is the minimum unit of the change in integers, we call the change *gradient* of the edge.

The two edges connecting the head node and the tail node of $v \in V$ in the original graph share the same gradient. Precisely, the gradient of the $(v, H, F) \to (v, T, F)$ and $(v, H, R) \to (v, T, R)$ is as follows:

$$\|c_v - (\#_N(v) + 1)\bar{c}\|^2 - \|c_v - \#_N(v)\bar{c}\|^2 \tag{5}$$

Also, we have converted an edge $e \in E$ into two edges, and they share the same gradient below:

$$\|c_e - (\#_E(e) + 1)\bar{c}\|^2 - \|c_e - \#_E(e)\bar{c}\|^2. \tag{6}$$

Suppose there is a cycle or a path from a source to a sink node in the double-stranded graph (Fig. S18d shows the latter case), and the sum of the gradient in it is negative. Then, if incrementing the copy numbers along this cycle or path indeed decreases the SSE (Eq. (2), we increment these copy numbers.

Note that there are corner cases in this algorithm. For example, if we select a cycle containing both $(v, H, F) \to (v, T, F)$ and $(v, H, R) \to (v, T, R)$, we traverse the v in the original chunk graph twice. In this case, the sum of the gradient in the cycle is not equal to the change of the SSE on the incrementation

along the cycle. Thus, it is unclear whether SSE would decrease by incrementing the copy numbers along this cycle.

We can avoid these cases with two heuristics. First, we confirm whether the SSE decreases before incrementing copy numbers to confirm that the SSE monotonously decreases during iterations. Second, if we can not confirm that the SSE does not decrease, we search for another path or cycle that does not contain these corner cases. Although there is no proof that we can find it, this search is usually successful in real datasets.

In the case of decrementing copy numbers, we can have almost the same algorithm with obvious modifications on the gradients, i.e., (5) and (6).

To find negative cycles or paths, although there is a dedicated algorithm ([10]), we employ the $O(|V||E|)$-time Bellman-Ford algorithm. Here, $|V|$ and $|E|$ are the number of nodes and edges, respectively. As the number of nodes is usually around thousands and the number of edges is almost linear to that of nodes, the Bellman-Ford algorithm is feasible in practice. Also, as we use 2000 bp long chunks by default, the copy numbers are usually less than 15, requiring at most 15 increments of the copy numbers along cycles or paths to reach an accurate estimation.

In summary, we employ the following four-step approach to accurately estimate the copy numbers of the chunks.

1. Align the chunks to reads (Fig. S17a).
2. Construct the chunk graph from the alignments (Fig. S17b).
3. Convert the chunk graph into the double-stranded graph (Fig. S18c).
4. Iteratively refine the estimated copy numbers by incrementing or decrementing the copy numbers along a path or cycle (Fig. S18d)

This four-step procedure considers not only the occurrences of the chunks in the reads but also the connections of the chunks on the target region to estimate the copy numbers accurately. The following section and the Method in the main text explain how to use this information in the clustering step.

6 Consensus and variant calling by a pair-hidden Markov Model

(In this section, let an alphabet Σ be $\{\mathtt{A},\mathtt{C},\mathtt{G},\mathtt{T}\}$. Also, a *string* s, a *read* r, and a *chunk* c are elements in Σ^*.)

6.1 A pair-hidden Markov model and basic algorithms

Consensus, i.e., polishing draft contigs, is one of the most crucial steps in the assembly as consensus errors directly affect the downstream analysis. Specifically, given that the average difference between haplotypes is around 0.1%, we need more than 99.9% accuracy to reliably infer the haplotype divergence.

Also, variant calling should be very accurate when we separate chunks into their copies which can be as similar as 99.9% (see Section 2.3 in the main text). This task is not trivial because the high error rates in the reads make it challenging to find true variants between haplotypes or segmental duplication.

Thus, we need sophisticated models and algorithms to take consensus and call variants. To this end, we use a pair-hidden Markov model (pHMM) and algorithms on it.

In this subsection, we define a pHMM and explain three algorithms that simulate how a DNA sequencer outputs a read from a chunk and compute the probability of observing a read. These algorithms are building blocks in the consensus and the variant calling.

A pair-hidden Markov model models a DNA sequencer. It has two major parameters modeling the state of a sequencer and the base we observe at each state.

Precisely, a pHMM has three states, M, D, and I, corresponding to the match, deletion, and insertion state to model the consecutive insertion and deletion errors in an ONT read. For example, the probability of observing a deletion error is smaller after a (mis-)match than after another deletion error. In other words, observing consecutive deletion errors is more likely than observing a deletion after a (mis-)match. To model these tendencies, a pHMM has $T_{s,s'}$ representing the transition probability from the state s to s':

$$T = \begin{pmatrix} T_{M,M} & T_{M,D} & T_{M,I} \\ T_{D,M} & T_{D,D} & T_{D,I} \\ T_{I,M} & T_{I,D} & T_{I,I} \end{pmatrix} \tag{7}$$

Now, a higher tendency of consecutive deletions can be modeled by setting $T_{M,D} < T_{D,D}$. Of course, the actual error patterns in the ONT reads are context dependent and more complex than the three-state model. Nonetheless, the more complex a model is, the more elaborate algorithm have to be developed for the model.[3] We assume that the three-state pHMM solves the trade-off between these two complexities.

We determine these transition probabilities based on alignments between chunks and reads. Shortly, we represent an alignment as an array of matches or mismatches (M), deletions (D), and insertions (I), and let an alignment between a read and a chunk be $L = L_0 \cdots, L_{N-1}$, where N is the length of the alignment, and $L_n \in \{\mathrm{M}, \mathrm{D}, \mathrm{I}\}$. Then, to estimate $T_{M,M}$, we divide the number of consecutive matches by the length of the alignments. Precisely[4],

$$T_{M,M} = \frac{|\{n \mid 0 \leq n < N-1, L_n = \mathrm{M}, L_{n+1} = \mathrm{M}\}|}{|\{n \mid 0 \leq n < N-1, L_n = \mathrm{M}\}|}$$

[3]Here, we use the word "complex" in an intuitive way. It may have something to do with "computational complexity" or other complexities, but, roughly speaking, we are talking about how it is easy to understand.

[4]We assume the alignment is longer than two, and there is at least one match.

It is easy to generalize the definition to more than two alignments and other values of T.

Each state has observation probabilities for generating a read from a chunk. The match state simulates the substitution errors of an ONT sequencer and outputs a base $x \in \Sigma$ depending on a base $y \in \Sigma$ of the chunk, and we denote this probability as $P_M(x|y)$.

Similarly, the insertion state simulates the base that ONT inserts in the reads, and we denote the probability of observing a base $x \in \Sigma$ at the insertion state as $P_I(x)$.

The deletion state always outputs the gap symbol $(-)$ or, equivalently, does not output anything.

We estimate these observation probabilities from alignments between reads and chunks. For example, suppose we have an alignment between a chunk and a read. To estimate $P_M(\mathtt{A}|\mathtt{A})$, we first count the number of matches between a base in the query and the adenine ($\mathtt{A}$) in the chunk. Specifically, let $\mathrm{Aln}(x|y)$ be the number of (mis-)matches in the alignment between the base x in the read and the base y in the chunk. Then,

$$P_M(\mathtt{A}|\mathtt{A}) = \frac{\mathrm{Aln}(\mathtt{A}|\mathtt{A})}{\sum_{x=\mathtt{A},\mathtt{C},\mathtt{G},\mathtt{T}} \mathrm{Aln}(x|\mathtt{A})}$$

Similarly, we estimate $P_I(x)$ by normalizing the count of the base x in the read at the insertion state.

A pHMM models a DNA sequencer, and we use it to generate a read from a chunk (Algorithm 1). Shortly, a pHMM changes its state and moves the position on the chunk that the sequencer reads during sequencing, which we call the *head* on the chunk. Initially, the state is on the match state, and the head is on the first base of the chunk. Then, the state moves among three states, and the head proceeds by one base every time we observe a base at the match or deletion state. Algorithm 1 iteratively makes transitions and observations until the head on the chunk leaves the last base.

We can use this simulation algorithm to evaluate a variant on a chunk such as SNP. Roughly speaking, we simulate ONT reads from the chunk to compute how many reads happen to support the variant due to sequencing errors, which enables us to quantitatively assess the variant. We will argue this procedure more deeply in Section 7.2.

We denote the probability, or the *likelihood*, that the above algorithm (Algorithm 1) generates a read r from a chunk c by a pHMM H as $L(r|c, H)$. Hereafter, we omit H from the notation when it is obvious from the context.

We can compute $L(r|c)$ by the forward algorithm (Algorithm 2) in $O(|r||c|)$ time. Shortly, this algorithm computes the probability that a pHMM outputs a state $s \in \{M, I, D\}$ and a read's prefix from a chunk's prefix. We denote this probability as $L(r_{\dots j}, s \mid c_{\dots i})$. Here, $r_{\dots j}$ is the prefix of r up to the $j-1$-th base, and $c_{\dots i}$ is the prefix of c up to the $i-1$-th base. Also, as the state s is before the condition $(|)$ in $L(r_{\dots j}, s \mid c_{\dots i})$, we can obtain $L(r|c)$ by summing up s over $L(r, s \mid c)$.

Another algorithm computes the probability that a pHMM generates a read's suffix from a chunk's suffix and a state $s \in \{M, I, D\}$, i.e., $L(r_{j\ldots}|c_{i\ldots}, s)$, which is called the backward algorithm (Algorithm 3). Note that the state s in the backward algorithms is in the condition in $L(r_{j\ldots}|c_{i\ldots}, s)$. Given the initial state is the match state M, $L(r|c)$ is the same as $L(r|c, M)$.

In the next section, we will explain an algorithm to compute the likelihood of r when introducing an edit operation to c by combining these two algorithms, which has been given by Chin ([1]).

Algorithm 1 Generating a read from a pair-HMM

Input: a pHMM and a chunk c
Output: A read r

```
s ← M                                              ▷ Start with the match state
i ← 0                                              ▷ The head on the chunk c
r ← the empty string
while i < |c| :                                    ▷ Until the pointer leaves the last base
    s ← select the next state from the probability T_{s,M}, T_{s,I}, and T_{s,D}
    if s = M :
        Generate a base x with the probability P_M(x|c_i) and push it to r   ▷ c_i is the i-th base of c.
        i ← i + 1
    else if s = I :                                ▷ If the state is the insertion state.
        Generate a base x with the probability P_I(x) and push it to r
    else
        i ← i + 1                                  ▷ A deletion state does not output base.
return r
```

6.2 Consensus and variant calling algorithm

As noted in the main text, we sample non-overlapping kilobase-scale subsequences from the ONT reads and call them "chunks." However, as ONT reads are error-prone, these chunks contain errors up to approximately 10%. We need to remove these errors to get an assembly with high accuracy. [5]

To formalize this problem, recall a pair-hidden Markov model simulates an ONT sequencer and computes the likelihood of generating a read r from a chunk c as $L(r|c)$. Maximizing the likelihood is expected to minimize the number of errors in the chunk.

Formally, for a set of reads R and a draft chunk c, we modify c to maximize the log-likelihood defined as (8).

$$\sum_{r \in R} \ln L(r|c) \tag{8}$$

[5] "removing errors in chunks" is a not well-defined problem because we do not **know** the ground truth for each chunk. In other words, there is no way to locate "errors" in a chunk.

Algorithm 2 The forward algorithm for a pair-HMM

Input: a pHMM, a read r, and a chunk c

Output: Likelihoods of outputting a state s and a read's prefix $r_{\dots j}$ from a chunk's prefix $c_{\dots i}$, $F_s[i][j] = L(r_{\dots j}, s|c_{\dots i})$

Initialize three $(|c|+1) \times (|r|+1)$ array F_M, F_D, F_I with 0
$F_M[0][0] \leftarrow 1$ ▷ The match state is the initial state.
for $i = 1, \cdots, |c|+1$ **:**
    $F_D[i][0] \leftarrow \sum_{s \in \{M,D,I\}} F_s[i-1][0] T_{s,D}$ ▷ $\sum_s$ runs over $\{M, D, I\}$ in other lines.
for $j = 1, \cdots, |r|+1$ **:**
    $F_I[0][j] \leftarrow \sum_s F_s[0][j-1] T_{s,I} P_I(r_{j-1})$ ▷ r_{j-1} is the $j-1$-th base of r.
for $i = 1, \cdots, |c|+1$, $j = 1, \cdots, |r|+1$ **:**
    $F_M[i][j] \leftarrow \sum_s F_s[i-1][j-1] T_{s,M} P_M(r_{j-1}|c_{i-1})$
    $F_D[i][j] \leftarrow \sum_s F_s[i-1][j] T_{s,D}$
    $F_I[i][j] \leftarrow \sum_s F_s[i][j-1] T_{s,I} P_I(r_{j-1})$
return F_M, F_I, F_D

Algorithm 3 The backward algorithm for a pair-HMM

Input: a pHMM, a read r, and a chunk c

Output: Likelihoods of outputting a read's suffix $r_{j\dots}$ from a chunk's suffix $c_{i\dots}$ and a state s, $B_s[i][j] = L(r_{j\dots}|c_{i\dots}, s)$

Initialize three $(|c|+1) \times (|r|+1)$ array B_M, B_D, B_I with 0
$B_{s \in \{M,D,I\}}[|c|+1][|r|+1] \leftarrow 1$ ▷ $\sum_s$ runs over $\{M, D, I\}$ in other lines.
for $i = |c|, \cdots, 0$ **:**
    $B_D[i][|r|+1] \leftarrow T_{D,D} B_D[i+1][|r|+1]$
    $B_I[i][|r|+1] \leftarrow T_{I,D} B_D[i+1][|r|+1]$
    $B_M[i][|r|+1] \leftarrow T_{M,D} B_D[i+1][|r|+1]$
for $j = |r|, \cdots, 0$ **:**
    $B_D[|c|+1][j] \leftarrow T_{D,I} P_I(r_j) B_I[|c|+1][j+1]$ ▷ r_j is the j-th base of r.
    $B_I[|c|+1][j] \leftarrow T_{I,I} P_I(r_j) B_I[|c|+1][j+1]$
    $B_M[|c|+1][j] \leftarrow T_{M,I} P_I(r_j) B_I[|c|+1][j+1]$
for $i = |c|, \cdots, 0$, $j = |r|, \cdots, 0$, $s = M, D, I$ **:**
    $B_s[i][j] \leftarrow T_{s,M} P_M(r_j|c_i) B_M[i+1][j+1] +$ ▷ from s to the match state.
        $T_{s,D} B_D[i+1][j] +$ ▷ from s to the deletion state.
        $T_{s,I} P_I(r_j) B_I[i][j+1]$ ▷ from s to the insertion state.
return B_M, B_I, B_D

Although there is no guarantee that we can reach the global maximum of (8), the algorithm we will explain in this section achieves more than 99.9% accuracy for 30-fold ONT reads.

Calling variants is similar to taking consensus. Suppose a chunk has an SNV position where the base in the maternal haplotype is `A` and that in the paternal is `C`. Then, if we change the base in the chunk at the SNV location from `C` to `A`, the likelihood of the reads from the maternal haplotype would increase because it would change a mismatch to a match at the SNV. Similarly, by changing the base from `A` to `C`, the likelihood of the reads from the paternal haplotype would increase. Conversely, if we find an edit operation that improves the likelihood on some, but not all, reads, it might represent a variation between haplotypes.

Overall, this section will compute how the likelihood of a read r changes if we apply an edit operation e to a chunk c, denoted as $P[r][i][e]$. In the rest of this section, we show how to compute $P[r][i][e]$ efficiently.

For simplicity, suppose we insert a new base `A` to the i-th position of the chunk and denote the modified chunk as $\hat{c}$. In other words, $\hat{c}$ is the concatenation of $c_{\dots i-1}$, `A`, and $c_{i\dots}$.

The critical observation is that we can decompose the generation of the read r from $\hat{c}$ by a pHMM as follows (Fig. S19):

1. Generate $r_{\dots j}$ and state s from $c_{\dots i}$. The forward algorithm computes this probability ($F_s[i][j]$).

2. Generate the j-th base of the read or a gap symbol $-$ at the newly introduced base `A`.

3. Generate $r_{j\dots}$ or $r_{j+1\dots}$ from $c_{i\dots}$ and the state D or M. The backward algorithm computes this probability ($B_D[i][j]$ or $B_M[i][j+1]$).

Note that $F_s[i][j] = L(r_{\dots j}, s|c_{\dots i})$ where $r_{\dots j}$ is the prefix of r up to the $j-1$-th base and $c_{\dots i}$ is defined similarly. Also, $B_M[i][j+1] = L(r_{j+1\dots}, |c_{i\dots}, M)$ where $r_{j+1\dots}$ is suffix of r from the $j+1$-th base and $c_{i\dots}$ is defined similarly.

If the pHMM generates a base of the read, there are $|r|$ cases, i.e., $r_0, \cdots, r_{|r|-1}$. Otherwise, the pHMM generates a gap symbol, and there are $(|r|+1)$ cases in total, i.e., before the j-th base for $j = 0, \cdots, |r|-1$, and after the last base.

Suppose we have computed F and B by the forward and backward algorithm beforehand. Then, if the added base (`A`) matches a base at some position j in the read, the total likelihood is

$$\sum_{s,j} F_s[i][j] T_{s,M} P_M(r_j|\mathtt{A}) B_M[i][j+1] \tag{9}$$

Otherwise, i.e., if the introduced base matches a gap symbol, the total likelihood is

$$\sum_{s,j} F_s[i][j] T_{s,D} B_D[i][j] \tag{10}$$

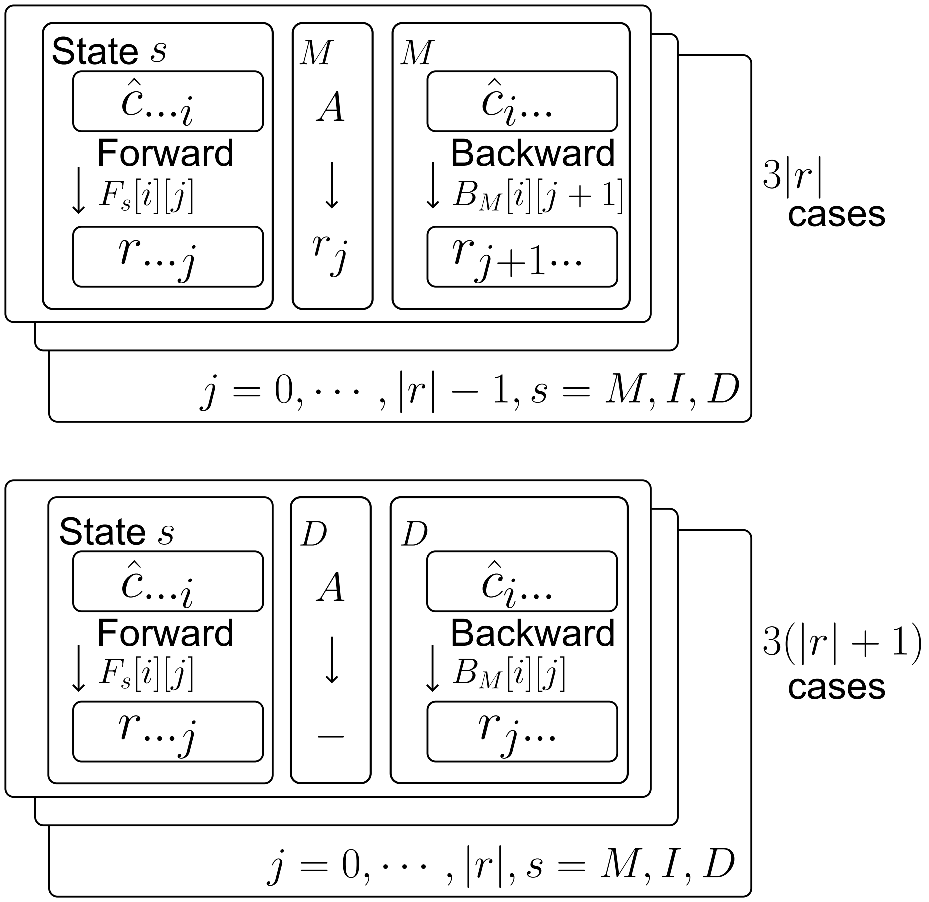

Figure S19: The decomposition of the process of generating a read r from an edited chunk $\hat{c}$. We can sum up the probabilities of these cases to get the probability of generating r from $\hat{c}$.

As we have already computed the B and F, it takes $O(|r|)$ to compute the likelihood $L(r|\hat{c})$. We can generalize this argument to substitutions and deletions for all positions in the chunk.

In summary, let $\hat{c}_{i,e}$ be the chunk after introducing an edit operation e at the i-th position of the chunk. $L(r|\hat{c}_{i,e})$ is the sum of (9) and (10). Given N reads, it takes $O(N|c||r|)$ time to compute the following value $P[r][i][e]$ for each read r, each position i in the chunk, and each edit operation e:

$$P[r][i][e] = \ln L(r|\hat{c}_{i,e}) - \ln L(r|c) \tag{11}$$

We call P the *perturbation matrix*, as it tells how the likelihood changes by a perturbation, or an edit operation, on a chunk.

How can we use the perturbation matrix to take a consensus from reads or, equivalently, to maximize the total log-likelihood $\sum_r \ln L(r|c)$?

To search for a nearly maximum value of the total log-likelihood, we sum up P over reads, i.e., we compute $\sum_r P[r][i][e]$. If this value is positive, the total likelihood increases by changing c into $\hat{c}_{i,e}$. Thus, we edit the chunk c by (i, e) until there are no (i, e) such that $\sum_r P[r][i][e] > 0$ holds. We regard the resulting sequence as the consensus sequence from the reads.

Also, we use the perturbation matrix P to call variants between the copies of a chunk. For example, suppose there is an SNV at the i-th base on the chunk, and we can represent the SNV as (i, e). Then, ideally, half of the reads support this SNV, i.e., half of the reads satisfy $P[r][i][e] > 0$ in the perturbation matrix P. Conversely, if we have a sufficient number of reads with $P[r][i][e] > 0$, it suggests that (i, e) is a variant between the copies of the chunk.

Formally, we sum up P over r but ignore negative values and denote it as V:

$$V[i][e] = \sum_r \max(P[r][i][e], 0) \tag{12}$$

$V[i][e]$ represents how much the total likelihood we can improve by splitting reads into two clusters based on the variants at (i, e). We regard a set of (i, e) giving large $V[i][e]$ as variants.

However, this naive criterion suffers from false positive variants. We will describe how to eliminate these false variants in the next section.

7 Filtering out false positive variants

The variant calling explained above does not consider two crucial properties of error-prone reads.

First, we should consider the biases in the base callers on the strand direction. Specifically, ONT sequencers introduce more errors in the forward strand on specific DNA sequences than in the reverse strand and vice versa. Thus, we sometimes observe a specific nucleotide that only occurs in the forward strand, which is often mistaken as a variant. We filter out the false positive variants

due to this *strand bias* by inspecting the perturbation matrix P in the forward and reverse strands separately.

Second, variant calling should consider the number of reads supporting a variant. For example, suppose 12 out of 60 reads on a chunk support a variant. This variant can be an artifact that occurred in 12 reads by chance due to sequencing errors. To rule out this possibility, we simulate reads by a pHMM to calculate the probability that we mistakenly regard an error as a variant.

By considering these two sources of false positive variants, the called variants from the perturbation become very accurate.

In the following two sections, we will argue how to determine whether a variant on a chunk is a false positive. For simplicity, we fix a chunk c, the reads R aligned to it, the position i, and the edit operation e at i-th position on the chunk. In other words, we hereafter denote the changes of the log-likelihood of each read $r \in R$ as $p_r = P[r][e][i]$ to make equations simple. In addition, we refer to the variant on the chunk represented as (i, e) as *the variant*.

7.1 Checking bias in the strand directions

If the copy number of the chunk c is two, and there is only one variant, (i, e), we separate the reads into two clusters, $\{r \mid p_r > 0\}$ and $\{r \mid p_r \leq 0\}$. By separating the reads into two copies in this manner, the log-likelihood improves by:

$$\sum_{r \in R} \max(p_r, 0) \tag{13}$$

If there is a bias in the strand direction, the difference between the value of (13) in the forward or reverse directions would be large. Let $\overrightarrow{R}$ be the set of reads aligned in the forward direction. We calculate the improvement of the likelihood on the forward strand as $\sum_{r \in \overrightarrow{R}} \max(p_r, 0)$. Similarly, by defining $\overleftarrow{R}$ as the reads aligned in the reverse direction, the improvement on the reverse strand is $\sum_{r \in \overleftarrow{R}} \max(p_r, 0)$. Then, we define the difference between them as d.

$$d = \sum_{r \in \overrightarrow{R}} \max(p_r, 0) - \sum_{r \in \overleftarrow{R}} \max(p_r, 0) \tag{14}$$

If there is a strand bias, d would be far from zero. We check this condition by a permutation test as follows.

First, we simulate a null distribution of d by shuffling the aligned direction and computing (14) two thousand times. Namely, at each repetition, we randomly sample $|\overrightarrow{R}|$ reads from R, denote it as S, and compute $\sum_{r \in S} \max(p_r, 0) - \sum_{r \notin S} \max(p_r, 0)$. If d is not in the 5%-95% interval of this null distribution, we regard the variant as a false positive. Two thousand times is usually sufficient to approximate the distribution.

7.2 Checking the number of supporting reads

There is another source of false positive variants besides the strand bias. Because of the high error rate, some reads often share the same error at the same position, which can be mistakenly regarded as a variant.

To discard these false positives, we estimate the probability p that a read supports a variant by chance. Specifically, we create three *mutated chunks*, each containing either an insertion, a deletion, or a substitution. Then, we generate N reads by Algorithm 1 from the chunk c and denote them as R'. For each mutated chunk $\hat{c}$, we count the fraction of generated reads with higher log-likelihoods on the mutated chunk than on the original chunk. Formally, we define the fraction p as follows:

$$p = \frac{|\{r \in R \mid L(r|\hat{c}) > L(r|c)\}|}{N} \tag{15}$$

($L(r|c)$ is the likelihood of generating a read r from a chunk c.)

We treat this probability p as the probability that a read supports an insertion, a deletion, or a substitution variant by chance. Thus, if we have M reads supporting the variant, we assume M follows the binomial distribution $\mathrm{Binom}(|R|, p)$. We filter out the variant if the p-value is above a significance level, $0.05/9|c|$, where 9 means the number of edit operations (four types of insertion, four types of substitution, and a deletion), and we use Bonferroni correction to keep the false positive rate low. We generate 1000 reads by default, as we find that generating 1000 reads is accurate and fast enough.

We remove most of the false variants by combining these two filtering functions with other naive filtering functions, such as removing variants in homopolymers. Empirically, as shown in Fig. 2 in the main text, we achieved almost optimal clustering by these filtering functions.

8 Read-vs-read alignments to polish clusterings

(See the Main text for the clustering algorithm.)

The previous section explained how to find variants based on the perturbation matrices. However, ONT reads are error-prone, and these errors can blur these variants in reads. As a result, these reads might not be assigned to the correct cluster. In this section, we will discuss how to resolve this issue.

Remember that a chunk can have adjacent chunks in the reads, and we carry out clusterings on these chunks independently. We can leverage the clusterings of chunks adjacent to the chunk to correct errors in the clustering on a chunk.

For example, suppose two chunks u and v are adjacent and share the same copy number K. Also, suppose these two chunks have the same set of reads R aligned to them. After the clustering, we have clusters of R on u and v. We denote these clusters, or partitions of R, as $R_1(u), \cdots, R_K(u)$ on u and $R_1(v), \cdots, R_K(v)$ on v. Then, if we were confident that the clustering on the chunk u is 100% accurate and the clustering on v is erroneous, we could discard

the clustering on v, and use u's clustering instead. In other words, we substitute $R_k(v)$ with $R_k(u)$ for each k.

However, in the actual cases, we are never confident that a clustering is 100% accurate, and u and v share only some of the reads R in general. Thus, the above argument does not work as-is fashion. In addition, we compare the clustering on only two chunks (u and v) in the above example. As reads are long and possibly have three or more chunks, it is desirable to consider clusterings as many as possible.

In summary, to remove errors in the clusterings, we need to handle three issues below:

- We need to know the accuracy of the clustering on a chunk.
- Adjacent chunks can have different sets of reads aligned to them.
- A read can be aligned to three or more chunks. It is desirable to consider all the clustering of these chunks.

To solve these issues, we align reads to each other not at the base level but at the chunk level. Fig. S20 gives the overview of our approach.

Intuitively, for the first issue, we devise a dedicated match score between two chunks for alignment so that we can measure the accuracy of clustering. Also, we allow alignments between reads to have gaps to consider the second issue. For the third issue, alignments can treat two or more chunks on a read.

These alignments between reads give *similarities* between reads, and we run a clustering algorithm on the similarity matrix to fine-tune clustering on the chunk (Fig. S20f).

Before explaining the detail of our method, we clarify our terminology. Specifically, we use the term "reads" in three meanings interchangeably so far:

- We have referred to the DNA sequences of ONT reads as "reads."
- We have called the region of a read aligned to a chunk "a read aligned to the chunk".
- We have used the word "read" to denote the array of these occurrences of the chunks in a read.

Although we have made our argument simple by using the word "read" flexibly, from now on, this flexibility will be confusing because a chunk can be aligned to two different positions of the same "read" (e.g., the chunk v appears twice in Read1 in Fig. S20b).

Thus, we will use the terms defined as follows (Fig. S21).

- A *chunk* is a DNA sequence.
- The *copies* of a chunk are DNA sequences in the target region similar to the chunk. For example, we have four copies of v in the two haplotypes in Fig. S21.

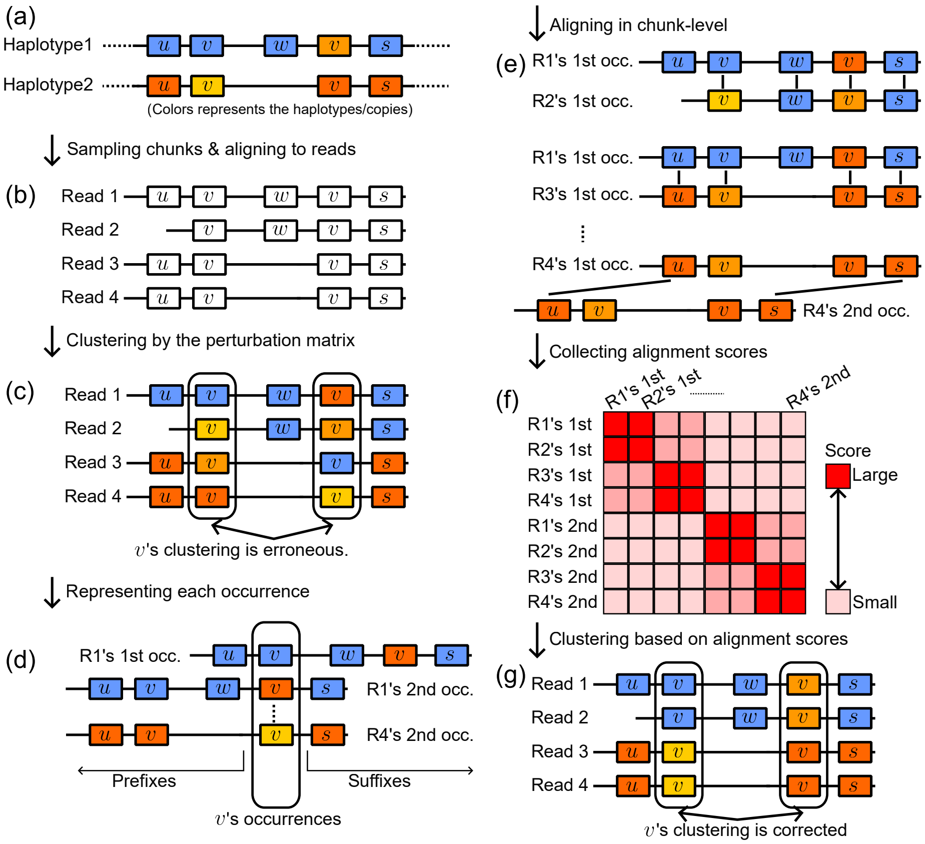

Figure S20: Schematic illustration of the read-vs-read alignments. (a): In this figure, we consider two haplotypes consisting of four chunks, u, v, w, and s. We color the chunks to distinguish the haplotypes and copies of these chunks. (b)-(c): Before clustering correction, we aligned these chunks to the reads and ran clusterings by the perturbation matrices on the chunks. Suppose that the clustering on v is erroneous due to the high sequencing error. (d): Before aligning the reads, we represent each of eight occurrences of v by a triple (the prefix, the occurrence, and the suffix). (e): We align these representations with each other by a dedicated match score (see Section 8.1). (f): The matrix of the alignment score. For example, the first occurrence of v in Read 1 and 2 aligns with a high alignment score. (g): The normalized spectral clustering on the matrix provides a new clustering of v, and we replace the old clustering with the new one.

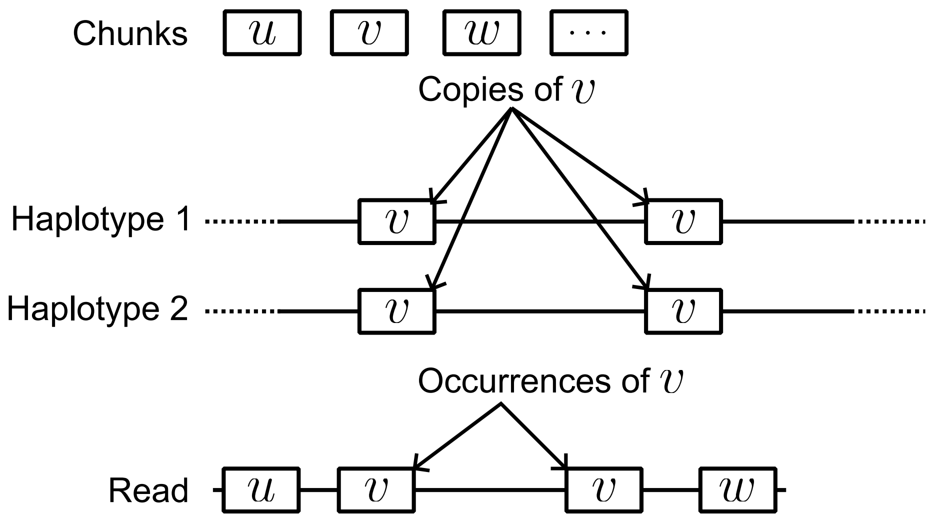

Figure S21: Definitions of occurrence and copies of a chunk.

- An *occurrence* of a chunk in a read is the sub-sequence of the read where we can align the chunk. For example, we have two occurrences of the chunk v in Read1 in Fig. S21.

We also use the term "read" to refer to the array of the occurrences of the chunks in a read. For example, Read in Fig. S21 is an array of occurrences of u, v, v, and w.

The definition of the alignment scheme is the most complicated part of this paper. Thus, we decompose the explanation into three parts:

1. Defining the match score between two occurrences of the chunks.
2. Defining the gap score and alignments between reads.
3. Converting alignment scores into a similarity score to correct errors.

8.1 Defining match score

To define a scoring scheme between reads, we first need to define the *match score* between two occurrences in the reads. In other words, what is the match score between w in Read1 and s in Read2 in Fig. S20e? How about between w in Read1 and w in Read2 in Fig. S20e? As explained in this section, we compute the probability that the two occurrences on the reads originate from the same genomic region. These two occurrences can "match" if this probability is greater than 0.5.

To compute this probability remember that chunks represent DNA sequences, and we have removed overlapping chunks (Section 4). Thus, two different chunks must originate from different regions, and the alignment score between the two

occurrences of them should be small. So, we define the match score between two occurrences of different chunks as $-\infty$. In other words, they never match in our alignment scheme regardless of the sequence similarity.

Next, we define the match score between two occurrences of the same chunk. To this end, let R be the occurrences of the chunk in the reads and the copy number of the chunk be K. We have run the clustering on this chunk by the perturbation matrix to partition these occurrences into K clusters, $R_1, \cdots, R_K$.

One obvious but not good approach for defining a match score is to use R_k directly. Namely, for r_1 and r_2 in R, we may define

$$\text{Match score}(r_1, r_2) = \begin{cases} 1 & \text{if } r_1 \in R_k \text{ and } r_2 \in R_k \text{ for some } k \\ 0 & \text{otherwise} \end{cases}$$

This match score fails to represent that different regions have different similarities. For example, suppose there are four chunks as in Fig. S22a. There are 1% differences between H1 and H2, while there are 0.1% differences between H2 and H3. Since the naive match score takes either 1 or 0, it fails to represent the similarities between these regions. In addition, this match score does not care about the errors in each occurrence.

To consider these two issues, we first relax the hard clustering, i.e., $R_1, \cdots, R_K$, to probabilistic representation. Namely, we will compute the probability that an occurrence $r \in R$ is in the k-th cluster based on the perturbation matrix P. This probability, denoted as $p_r[k]$, provides a way to define the match score.

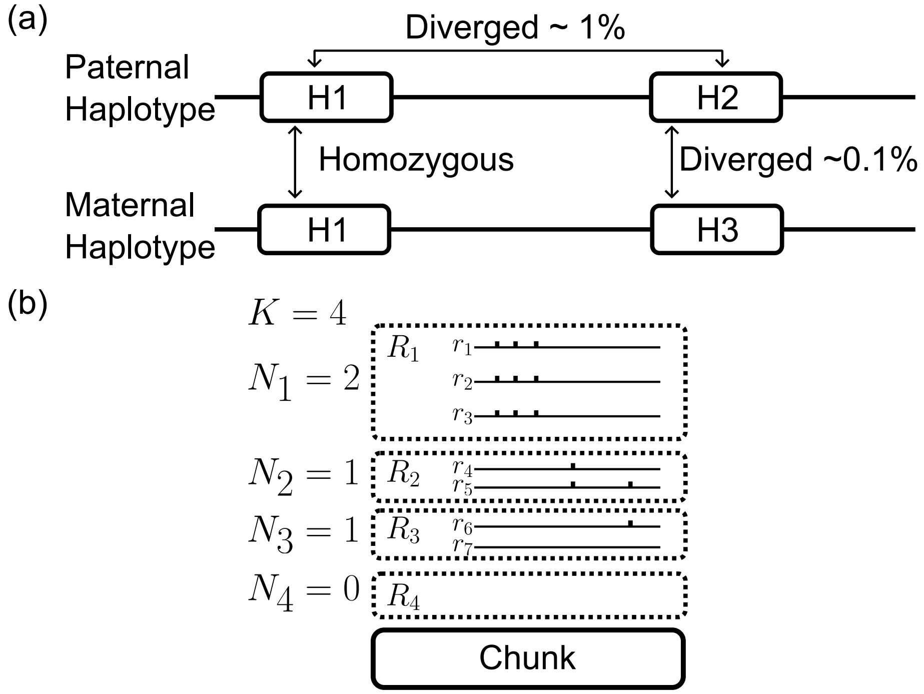

Figure S22: A schematic illustration of the match score. (a): If a chunk's copy number is four, four regions in the underlying genome correspond to these copies. Note that these regions can be homozygous. (b): The example of the perturbation matrix (Eq. (16)). The vertical ticks in the occurrences represent the variants. K is the copy number, and $N_k, k = 1, \cdots, 4$ is the estimated copy number of each cluster.

For the reader's understanding, we give a concrete example of the occurrences, a perturbation matrix, and a partition of them (Fig. S22b).

To begin with, remember that a perturbation matrix represents how the log-likelihood of a read changes when we apply an edit operation to the chunk. We simplify these changes and use $1, -1$, and 0 to represent the case when the log-likelihood increases, decreases, or does not change, respectively.

Under this simplification, our example consists of seven occurrences $R = \{r_1, \cdots, r_7\}$ and the perturbation matrix defined as Eq. (16). Here, for simplicity, we assume the set of selected variants U has five pairs of (i, e), and we regard U as $\{1, 2, 3, 4, 5\}$. [6]

Further, suppose we have partitioned these occurrences into three clusters, $R_1 = \{r_1, r_2, r_3\}$, $R_2 = \{r_3, r_4\}$, and $R_3 = \{r_6, r_7\}$. We visualize this example in Fig. S22b.

[6] In the actual implementation, we have nine types of edit operations and convert $P(i, e)$ into $9i + e$, making $P[r][i][e]$ to be $|R| \times 9L$-dimensional matrix.

$$P = \begin{pmatrix} P[r_1] \\ P[r_2] \\ P[r_3] \\ P[r_4] \\ P[r_5] \\ P[r_6] \\ P[r_7] \end{pmatrix} = \begin{pmatrix} 1 & 1 & 1 & -1 & -1 \\ 1 & 1 & 1 & -1 & -1 \\ 1 & 1 & 1 & -1 & -1 \\ -1 & -1 & -1 & 1 & 0 \\ -1 & -1 & -1 & 1 & 1 \\ -1 & -1 & -1 & -1 & 1 \\ -1 & -1 & -1 & -1 & 0 \end{pmatrix} \tag{16}$$

Intuitively, the first three columns of the perturbation matrix Eq. (16) represent the variant separating R_1 from other clusters, the fourth column characterizes R_2, and the fifth column separates R_2 and R_3 from R_1. Also, as errors in the fourth occurrence r_4 erased the fifth variant (i.e., $P[r_4][5] = 0$), the assignment of r_4 is less unreliable than the assignment of the fifth occurrence, r_5. We integrate these intuitions into $p_r[k]$ by first expressing the difference between clusters $R_1, \cdots, R_K$ and then representing the sequencing errors.

To capture the difference between clusters, we sum up the perturbation matrix P over R_k. If the value, $\sum_{r \in R_k} P[r][i][e]$, is positive, the variant (i, e) increases the total log-likelihood in the k-th cluster.

In the running example,

$$\begin{matrix} R_1 \\ R_2 \\ R_3 \end{matrix} \begin{pmatrix} P[r_1] + P[r_2] + P[r_3] \\ P[r_4] + P[r_5] \\ P[r_6] + P[r_7] \end{pmatrix} = \begin{pmatrix} 3 & 3 & 3 & -3 & -3 \\ -2 & -2 & -2 & 2 & 1 \\ -2 & -2 & -2 & -2 & 1 \end{pmatrix} \tag{17}$$

The first, second, and third variants increase the log-likelihood in R_1.

We define these (i, e) as U_k and denote the chunk after introducing all the operations in U_k to c as $\hat{c}_{U_k}$. [7] Precisely,

$$U_k = \{(i, e) \in U \mid \sum_{r \in R_k} P[r][i][e] > 0\} \tag{18}$$

$$\hat{c}_{U_k} = \text{sequence after applying all elements in } U_k \text{ to } c. \tag{19}$$

In the running example, $U_1 = \{1, 2, 3\}$, $U_2 = \{4, 5\}$, and $U_3 = \{5\}$.

By writing $z_r = k$ to mean r is in the k-th cluster, we calculate the probability that the occurrence r is in the k-th cluster, $p_r[k]$, as follows:

$$\begin{aligned} p_r[k] &= Pr\{z_r = k \mid r, c, \hat{c}_{U_1}, \cdots, \hat{c}_{U_K}\} && (20) \\ &= Pr\{r \mid z_r = k, c, \hat{c}_{U_1}, \cdots, \hat{c}_{U_K}\} \frac{Pr\{z_r = k \mid c, \hat{c}_{U_1}, \cdots, \hat{c}_{U_K}\}}{Pr\{r \mid c, \hat{c}_{U_1}, \cdots, \hat{c}_{U_K}\}} && (21) \\ &\propto Pr\{r \mid z_r = k, c, \hat{c}_{U_1}, \cdots, \hat{c}_{U_K}\} Pr\{z_r = k \mid c, \hat{c}_{U_1}, \cdots, \hat{c}_{U_K}\} && (22) \\ &\propto Pr\{r \mid z_r = k, c, \hat{c}_{U_1}, \cdots, \hat{c}_{U_K}\} && (23) \\ &= Pr\{r \mid \hat{c}_{U_k}\} && (24) \end{aligned}$$

[7] This might not be a valid sequence, as U_k can contain two operations at the same position – two substitutions at the same base of the chunk. However, we can avoid this issue by forcing each element (i, e) in U_k to be separated by at least D bases.

Here, we use Bayes' theorem to obtain Eq. (21), drop a term independent from k to obtain Eq. (22), assume $Pr\{z_r = k \mid c, \hat{c}_{U_1}, \cdots, \hat{c}_{U_K}\}$ is a uniform distribution to obtain Eq. (23), and use $z_r = k$ to obtain Eq. (24).

Thus, we need $Pr\{r \mid \hat{c}_{U_k}\}$, the likelihood of r from $\hat{c}_{U_k}$, to compute the posterior probability, $p_r[k]$.

To this end, remember that $P[r][i][e]$ is how the log-likelihood changes when applying the variant (i, e) to the chunk c. Thus, we can approximate the change of the log-likelihood when applying all the operations in U_k to the chunk c by summing up $P[r][i][e]$ over (i, e) in U_k. We denote this value as $l(r|\hat{c}_{U_k})$. Formally,

$$l(r|\hat{c}_{U_k}) = \sum_{(i,e)\in U_k} P[r][i][e] \tag{25}$$

As a result, we approximate the log-likelihood of r in the k-th cluster. Precisely, given that the likelihood of an occurrence r from the chunk is defined as $L(r|c)$ (Section 6.1), $\ln Pr\{r \mid \hat{c}_{U_k}\} = \ln L(r|\hat{c}_{U_k}) \approx \ln L(r|c) + l(r|\hat{c}_{U_k})$.

In the running example, we have the following vector for r_1.

$$\begin{pmatrix} l(r_1|\hat{c}_{U_1}) \\ l(r_1|\hat{c}_{U_2}) \\ l(r_1|\hat{c}_{U_3}) \end{pmatrix} = \begin{pmatrix} P[r_1][1] + P[r_1][2] + P[r_1][3] \\ P[r_1][4] + P[r_1][5] \\ P[r_1][5] \end{pmatrix} = \begin{pmatrix} 3 \\ -2 \\ -1 \end{pmatrix} \tag{26}$$

Similarly, for r_4 and r_5, we have

$$\begin{pmatrix} l(r_4|\hat{c}_{U_1}) \\ l(r_4|\hat{c}_{U_2}) \\ l(r_4|\hat{c}_{U_3}) \end{pmatrix} = \begin{pmatrix} -3 \\ 1 \\ 0 \end{pmatrix}, \begin{pmatrix} l(r_5|\hat{c}_{U_1}) \\ l(r_5|\hat{c}_{U_2}) \\ l(r_5|\hat{c}_{U_3}) \end{pmatrix} = \begin{pmatrix} -3 \\ 2 \\ 1 \end{pmatrix} \tag{27}$$

Now, we are ready to compute $p_r[k]$, the probability that the occurrence r is in the k-th cluster.

$$\begin{aligned} p_r[k] &\propto Pr\{r \mid \hat{c}_{U_k}\} && (28) \\ &\approx \exp\left(\ln L(r|c) + l(r|\hat{c}_{U_k})\right) && (29) \\ &\propto \exp l(r|\hat{c}_{U_k}) && (30) \end{aligned}$$

Here, we remove $L(r|c)$ to obtain Eq. (30) because it does not depend on k.

We call $p_r[k]$ the *posterior probability* that the occurrence r is in the k-th cluster.

In the example (Eq. (16), Fig. S22), the posterior probability of the first occurrence r_1 is as follows:

$$\begin{pmatrix} p_{r_1}[1] \\ p_{r_1}[2] \\ p_{r_1}[3] \end{pmatrix} = \begin{pmatrix} e^3 \\ e^{-2} \\ e^{-1} \end{pmatrix} / \left(e^3 + e^{-2} + e^{-1}\right) \approx \begin{pmatrix} 0.97 \\ 0.01 \\ 0.02 \end{pmatrix} \tag{31}$$

Likewise, we can compute the posterior probabilities of r_4 and r_5.

$$\begin{pmatrix} p_{r_4}[1] \\ p_{r_4}[2] \\ p_{r_4}[3] \end{pmatrix} \approx \begin{pmatrix} 0.01 \\ 0.72 \\ 0.27 \end{pmatrix}, \begin{pmatrix} p_{r_5}[1] \\ p_{r_5}[2] \\ p_{r_5}[3] \end{pmatrix} \approx \begin{pmatrix} 0.00 \\ 0.73 \\ 0.27 \end{pmatrix}, \tag{32}$$

At the beginning of this section, we argued that r_4 should be less reliable than r_5. As we expected, while the probability that r_4 is in the first cluster R_1 is $p_{r_4}[1] = 1\%$, there is almost no chance that r_5 is in R_1 (i.e., $p_{r_5}[1] \approx 0$).

Now, we can compute the probability that two occurrences originate from the same clusters. Specifically, the posterior probability of r_2 is as follows:

$$\begin{pmatrix} p_{r_2}[1] \\ p_{r_2}[2] \\ p_{r_2}[3] \end{pmatrix} \approx \begin{pmatrix} 0.97 \\ 0.01 \\ 0.02 \end{pmatrix} \tag{33}$$

Thus, the probability r_1 and r_2 originate from the same cluster is the inner product of the posteriors:

$$\begin{pmatrix} p_{r_1}[1] \\ p_{r_1}[2] \\ p_{r_1}[3] \end{pmatrix} \cdot \begin{pmatrix} p_{r_2}[1] \\ p_{r_2}[2] \\ p_{r_2}[3] \end{pmatrix} = \sum_{k=1}^{3} p_{r_1}[k] p_{r_2}[k] = 0.97^2 + 0.01^2 + 0.02^2 \approx 0.94 \tag{34}$$

However, as depicted in Fig. S22a, there are two copies of H1, and two occurrences can come from these two different copies of H1. Thus, this probability differs from the probability that these two occurrences originate from **the same genomic region**. We need to consider the copy numbers of these clusters.

Specifically, the chance that two occurrences in the H1 share the same genomic region is 1/2. Therefore, we can calibrate the probability by dividing $p_{r_1}[1]p_{r_2}[1]$ by two. The correct probability is $0.97^2/2+0.01^2/1+0.02^2/1 \approx 0.47$.

To generalize this idea, we denote $C_1, \cdots, C_K$ as the number of occurrences in the clusters, i.e., $C_k = |R_k|$. Also, let $\bar{c}$ be the average haploid coverage, which is determined by dividing the total input bases by two times the size of the target region [8]. Then, we estimate the copy numbers of clusters as $N_1, \cdots, N_K$ by minimizing the mean squared error defined below such that $\sum_k N_k = K$ (Fig. S22b).

$$\sum_k \|\bar{c}N_k - C_k\|^2 \tag{35}$$

We obtain the optimal solution by a greedy algorithm (Algorithm 4).

Now, we have all materials needed to define the match score between two occurrences of the same chunk. Suppose we have the posterior probabilities of two occurrences of the same chunk, $p = (p_1, \cdots, p_K)$ and $q = (q_1, \cdots, q_K)$. These occurrences are on the same genomic region if they are in the same cluster (with a probability of $p_k q_k$ for each k), the cluster has at least one copy in the genome ($N_k > 0$), and they are in an identical copy (with a probability of $1/N_k$). We denote this probability as the *match probability* a:

[8] $T/2|G|$, where T is the total bases, and $|G|$ is the size of the target region.

Algorithm 4 Copy number (N_k) estimation for a clustering

Input: The size of the clusters $C_1, \cdots, C_K$ and the haploid coverage $\bar{c}$
Output: The copy number of clusters, $N_1, \cdots, N_K$

Initialize $N_1, \cdots, N_K$ as 0
for K times **:**
Select k that reduces the mean squared error the most.
  $k \leftarrow \arg\max_k \|\bar{c}N_k - C_k\|^2 - \|\bar{c}(N_k+1) - C_k\|^2$
  $N_k \leftarrow N_k + 1$
return $N_1, \cdots, N_K$

$$a(p, q) = \sum_{k=1, N_k \neq 0}^{K} \frac{p_k q_k}{N_k} \tag{36}$$

We convert this match probability to *match score* by mapping the probability a to the log-odds-ratio, $\ln \frac{a}{1-a}$. [9]

To see the rationale behind this mapping, suppose $a(p,q) = 0.5$. Then, a is equal to $1 - a$. Thus, the probability that two occurrences originate from the same genomic region is the same as the probability that they do not. In this case, the match score is zero ($\ln \frac{0.5}{1-0.5} = 0$). Also, the log-odds-ratio is positive if and only if $a > 0.5$. Thus, it gives a reasonable match score because it prefers match probabilities more than 0.5.

In the running example, the match probability and score between r_4 and r_5 are:

$$a(p_{r_4}, p_{r_5}) = \frac{0.01 \cdot 0.00}{2} + \frac{0.72 \cdot 0.73}{1} + \frac{0.27 \cdot 0.27}{1} \approx 0.6$$
$$\ln \frac{a(p_{r_4}, p_{r_5})}{1 - a(p_{r_4}, p_{r_5})} \approx 0.4$$

In contrast, as the match probability between r_1 and r_5 is as small as 0.01, the match score is $\ln \frac{0.01}{0.99} \approx -4.3 < 0$.

As expected, the match score prefers the match among the same cluster, i.e., r_4 and r_5, than the match between different clusters, i.e., r_1 and r_5.

In summary, the match score between two occurrences (r_1 and r_2) is defined as follows:

$$\text{Match score}(r_1, r_2) = \begin{cases} -\infty & \text{if they represent two different chunks} \\ \ln \frac{a}{1-a} & \text{otherwise} \end{cases} \tag{37}$$

In the next section, we represent gaps in the reads and derive the complete alignment scheme to correct errors in the clusterings.

[9] Note that if $a = 0$ or $a = 1$, the log-odds-ratio is undefined. To avoid $a = 0$, we convert a to $\max(a, 10^{-20})$. Similarly, to avoid $a = 1$, we convert a to $\min(a, 1 - 10^{-20})$

8.2 Define gaps scores and alignments between two reads

Because of the high error rate in the reads, we sometimes can not align a chunk to a read, making a "gap" in the reads (Fig. S23a). Thus, to consider gaps caused by highly erroneous regions, we need to allow gaps in chunk-level alignments.

In contrast, suppose the genome has a large heterozygous insertion (Fig. S23b). Chunks derived from this insertion appear only in one haplotype and create large insertions in the reads at the chunk level.

These two types of gaps are different because we want to allow only the first case of these two. Thus, we introduce the gap open penalty G and the gap extension penalty E. By setting G nearly zero and E much smaller than G, we allow one-length gaps caused by sequencing errors and inhibit large gaps caused by structural variants. After testing several parameters, we set $G = -0.5$ and $E = -100$ as default values.

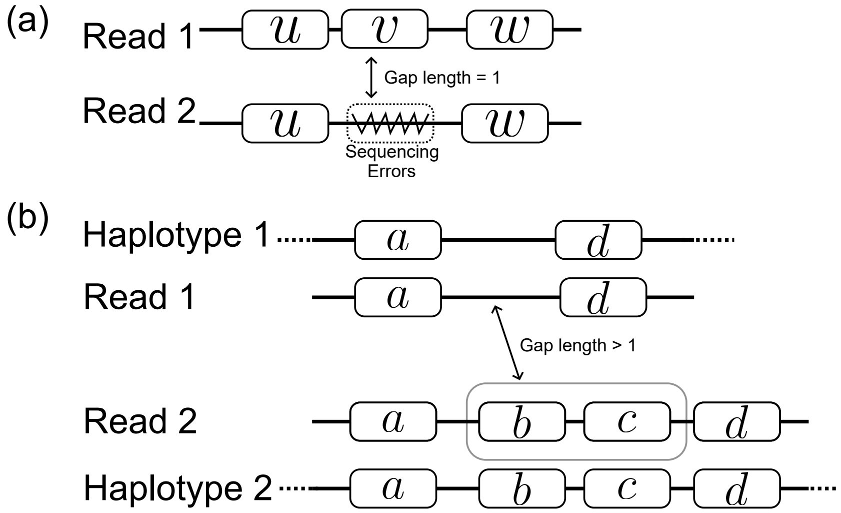

Figure S23: Schematic illustration of the two types of gaps in the alignments at chunk-level. (a): The gap created by high sequencing errors in the reads. The chunk v is absent from the second read. (b): The gap created by a large structural variant between the haplotypes. The two chunks (b and c) are absent from the first read. We only allow alignments from the first case by setting the gap extension penalty much smaller than the open penalty.

The last thing to consider is the multiple occurrences of the same chunk in the same read. Specifically, suppose a read has two occurrences of the same chunk (e.g., Read1 in Fig. S20 has the chunk v twice). To distinguish these two occurrences, we first locate the position of the occurrence in the reads. Then, we split the reads into three components – the prefix before it, the occurrence itself, and the suffix after it. For example, we convert two occurrences of v in Read 1 in Fig. S20 as follows:

1. The first occurrence is converted to the prefix (u), the occurrence itself, and the suffix (w, v, s).

2. The second occurrence is converted to the prefix (u, v, w), the occurrence itself, and the suffix (s).

Given two of these representations, we align the prefixes to each other, the occurrence to each other, and the suffixes to each other by the alignment scheme (Eq. (37), G, and E).

For given N occurrences of a chunk, these alignments provide an $N \times N$-dimension matrix A, where the (i, j)-element is the alignment score between the i-th and the j-th occurrences. In the next section, we will discuss how to convert this matrix A into a similarity matrix to carry out clustering.

8.3 Convert an alignment score into a similarity score

The final step is to find the clusters from the alignment scores between reads.

To this end, remember that we define the match score as the log-odds-ratio of the alignment probability. Further, we assume that the alignment score between two reads is the log-odds-ratio of the *overlapping probability.* Formally, we interpret the matrix A as

$$A[i][j] = \text{alignment score between } r_i \text{ and } r_j \tag{38}$$

$$= \ln \frac{P\{r_i \text{ aligns } r_j\}}{1 - P\{r_i \text{ aligns } r_j\}} \tag{39}$$

We solve this equation to get a $N \times N$-dimension similarity matrix S.

$$S[i][j] = P\{r_i \text{ aligns } r_j\} = \frac{1}{1 + \exp(-A[i][j])} \tag{40}$$

As illustrated in Fig. S24, this conversion maps large alignment scores to near one and small scores to near zero.

Finally, we run the spectral clustering on S (Algorithm 5) and replace the original clustering $R_1, \cdots, R_K$ with them. We select the normalized spectral clustering because it handles the similarity matrix well. See ([8]) for further rationale.

In summary, we correct errors in the clusterings in four steps.

1. For each chunk, we convert occurrences R of the chunk into posterior probabilities by Eq. (30).

2. For each chunk v,

   (a) We represent each occurrence of the chunk v in the reads into three components – the prefix up to it, the occurrence itself, and the suffix from it.

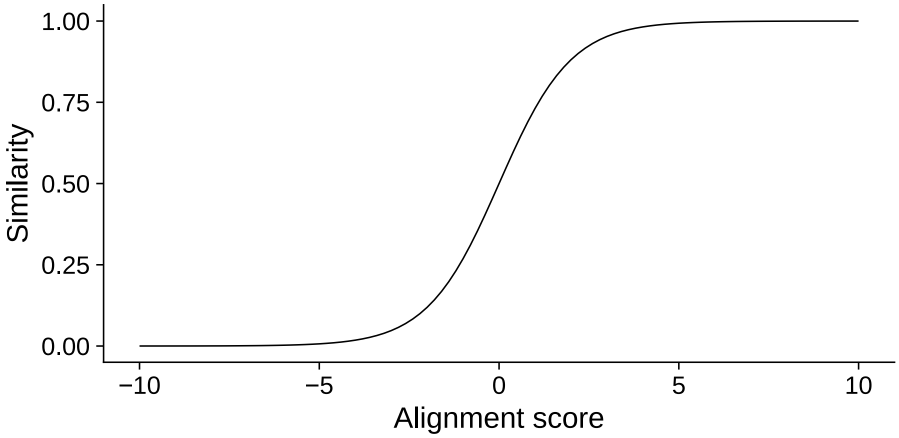

Figure S24: The function to convert the alignment score into similarity.

Algorithm 5 The spectral clustering on a similarity matrix.

Input: $N \times N$ similarity matrix S and an eigenvalue threshold t(=0.25 by default)
Output: Clustering of N elements

Let D be a diagonal matrix with $D_{i,i} = \sum_j S_{i,j}$ ▷ Row sum
$L \leftarrow I - D^{1/2} S D^{1/2}$
$(v_1, \cdots, v_k) \leftarrow$ eigenvectors with eigenvalue smaller than t. ▷ There are k clusters
$V \leftarrow$ Combine $v_1, \cdots, v_k$ to make an $N \times k$ matrix.
Regard rows of V as k-dimensional feature vectors for elements.
Clustering these feature vectors by k means clustering.

(b) We align them with each other by the scoring scheme defined by Eq. (37), $G(=-0.5)$, and $E(=-100)$ to get alignment scores A.

(c) We convert the matrix of alignment score A into the similarity matrix S by Eq. (40).

(d) We carry out the spectral clustering on S to obtain new clustering $R_1, \cdots, R_K$, and replace the original clustering of v with them.

The posterior probabilities consider the similarities between clusters and errors in the reads. Also, the chunk-level alignments consider the long-range information given by the reads. Thus, this approach utilizes the sequence and structural information that the reads give us.

9 The serializing algorithm on the partially phased graph

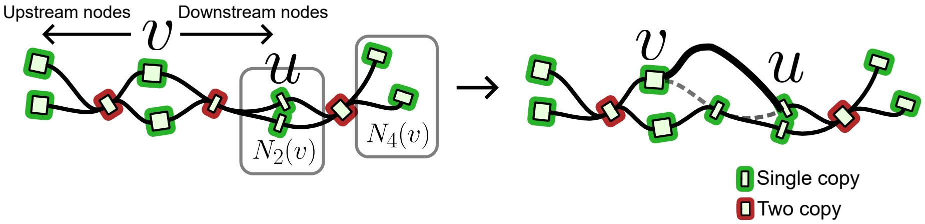

Figure S25: A schematic illustration of the graph simplification. The two graph boxes show the $N_2(v)$ (right). If u and v are foci for each other, we remove a path and add an edge between u and v (left).

The clustering aims to phase the chunks to obtain a fully phased assembly. However, there are long homozygous regions, i.e., regions without any variants. The clustering based on variants can not separate the copies of a chunk in these regions. Thus, we need an additional algorithm to solve these completely homozygous regions to produce a fully phased assembly.

Here, very long reads are helpful. Intuitively, if a read spans a homozygous region and connects phased regions, we can merge them. To handle a few remaining errors in clusterings, we develop merging criteria based on a statistical method.

We first construct a graph called *phased chunk graph* with the clustering information on the chunks in an analogous way to the *chunk graph* in Section 5.

Precisely, suppose that we have partitioned each chunk c into its K_c copies. Then, we have K nodes $(c,1), \cdots, (c,K)$ in the graph for each chunk c. We call two nodes (c,k) and (c',k') *adjacent* if they satisfy the following four conditions:

- There is a read r such that c and c' are aligned to it.

- There are no other chunks between c and c' in r.
- The occurrence of c in r is in the k-th cluster in the c's clustering.
- The occurrence of c' in r is in the k'-th cluster in the c''s clustering.

Like the chunk graph in Section 5, we introduce H and T to represent the first (head) and last (tail) bases of a copy of a chunk.

By using these components, we define a *phased chunk graph* $G = (V, E)$ as follows:

- The node V is the union of $\{c_1, \cdots, c_K\}$ for each chunk c.
- The edge $E \subseteq (V \times \{H, T\}) \times (V \times \{H, T\})$ contains an edge between (v, p) and (u, q) if and only if v and u are adjacent and the p position of v and the q of u are connected by a read.
- Rigorously, we should define $V \times \{H, T\}$ as the nodes, but for simplicity, we refer to the set of the copies of the chunks as V.

On a phased chunk graph, we estimate the copy numbers of each node $v = (c, k)$ using the same algorithm explained in Section 5. We define the upstream nodes of a node v in the graph as the nodes that are reachable from the head position of v (Algorithm 6). Also, the downstream nodes of v are the nodes reachable from the tail position of v.

Algorithm 6 Enumerating reachable nodes from a given node

Input: Phased chunk graph G, node v on G, start position p (= Head or Tail), and the max distance $D \in \mathbb{N}$

Output: The reachable nodes $N(v) = N_1(v), \cdots, N_D(v)$ from the position p of v with distance $d \leq D$.

$N_1(v), \cdots, N_D(v) \leftarrow ([\ ], [\ ], \cdots, [\ ])$ ▷ Initialize by the empty arrays.
$N_0(v) \leftarrow [(v, p)]$
for $d = 1, \cdots, D$ **:**
  for $(x, s) \in N_{d-1}(v)$ **:**
    for each edge $e = (x, s) \leftrightarrow (y, t)$ in G **:**
      if t is Head **:** ▷ Go to (y, t) then to the opposite side of y
        Push (y, Tail) to $N_d(v)$.
      else
        Push (y, Head) to $N_d(v)$.
Remove the second element, $(\_, p)$, from $N_1(v) \cdots, N_D(v)$.
return $N_1(v), \cdots, N_D(v)$

Then, let v be a node adjacent to a homozygous node, and suppose we are finding the node downstream of v that we can merge with v.

To this end, let $N_d(v)$ be the set of nodes at a distance d downstream of v (Fig. S25), $N = |N_d(v)|$, and R be the number of reads that contain v and

at least one of the nodes in $N_d(v)$. Our method aims to check whether we can merge v and a node in $N_d(v)$.

Suppose there are R_u reads containing both v and $u \in N_d(v)$. If there were no errors in the clustering, we could span from v to u whenever R_u is greater than zero, i.e., whenever there are spanning reads. However, in reality, there are a small number of errors. Thus, we check whether R_u is statistically significant by using two binomial distributions $\mathrm{Binom}(R_u|R,p)$ with different values of p.

The first distribution represents the null hypothesis where a read from v reaches a node in $N_d(v)$ with an equal probability, i.e., R_u follows the binomial distribution with parameter $p = \frac{1}{N}$. Under this model, the likelihood of seeing R_u reads at $u \in N_d(v)$ is as follows:

$$P_{\text{null}}(R_u|R,N) = \binom{R}{R_u}(\frac{1}{N})^{R_u}(1-\frac{1}{N})^{R-R_u} \tag{41}$$

The second distribution represents the alternative hypothesis where almost all the reads passing v also pass u. Precisely, let ϵ be a small value, 0.1 by default, and define the likelihood as follows:

$$P_{\text{alt}}(R_u|R,N) = \binom{R}{R_u}(1-\epsilon)^{R_u}\epsilon^{R-R_u} \tag{42}$$

We compare these two models by log-likelihood and denote it as $L_d(u|v) = \ln P_{\text{alt}} - \ln P_{\text{null}}$. We define the node and the distance giving the maximum $L_d(u|v)$ as the *focus* of v:

$$\text{focus}(v) = \arg\max_{d,u} L_d(u|v) \tag{43}$$

To span the homozygous region from v with minimum errors, we search a distance d and a node u such that

$$\text{focus}(v) = (d,u) \tag{44}$$
$$\text{focus}(u) = (d,v) \tag{45}$$

If there is any such (d,u), we eliminate an arbitrary path and draw an edge between v and u. We also decrement the copy number of the node in the eliminated path by one.

This simple statistical method accurately separates long homozygous regions by utilizing very long ONT reads and enables us to produce fully phased assemblies.

References

[1] Chen-Shan Chin, David H. Alexander, Patrick Marks, Aaron A. Klammer, James Drake, Cheryl Heiner, Alicia Clum, Alex Copeland, John Huddleston, Evan E. Eichler, Stephen W. Turner, and Jonas Korlach. Nonhybrid,

finished microbial genome assemblies from long-read smrt sequencing data. *Nature Methods*, 10(6):563–569, Jun 2013.

[2] Erich D. Jarvis et al. Semi-automated assembly of high-quality diploid human reference genomes. *Nature*, 611(7936):519–531, Nov 2022.

[3] Mikhail Kolmogorov et al. Assembly of long, error-prone reads using repeat graphs. *Nature Biotechnology*, 37(5):540–546, May 2019.

[4] Peter Krusche, Len Trigg, Paul C. Boutros, Christopher E. Mason, Francisco M. De La Vega, Benjamin L. Moore, Mar Gonzalez-Porta, Michael A. Eberle, Zivana Tezak, Samir Lababidi, Rebecca Truty, George Asimenos, Birgit Funke, Mark Fleharty, Brad A. Chapman, Marc Salit, Justin M. Zook, the Global Alliance for Genomics, and Health Benchmarking Team. Best practices for benchmarking germline small-variant calls in human genomes. *Nature Biotechnology*, 37(5):555–560, May 2019.

[5] Heng Li. Minimap2: pairwise alignment for nucleotide sequences. *Bioinformatics*, 34(18):3094–3100, 05 2018.

[6] Heng Li, Jonathan M. Bloom, Yossi Farjoun, Mark Fleharty, Laura Gauthier, Benjamin Neale, and Daniel MacArthur. A synthetic-diploid benchmark for accurate variant-calling evaluation. *Nature Methods*, 15(8):595–597, Aug 2018.

[7] Jyun-Hong Lin et al. LongPhase: an ultra-fast chromosome-scale phasing algorithm for small and large variants. *Bioinformatics*, 38(7):1816–1822, 02 2022.

[8] Ulrike von Luxburg. A tutorial on spectral clustering, 2007.

[9] Justin Wagner, Nathan D. Olson, Lindsay Harris, Ziad Khan, Jesse Farek, Medhat Mahmoud, Ana Stankovic, Vladimir Kovacevic, Byunggil Yoo, Neil Miller, Jeffrey A. Rosenfeld, Bohan Ni, Samantha Zarate, Melanie Kirsche, Sergey Aganezov, Michael C. Schatz, Giuseppe Narzisi, Marta Byrska-Bishop, Wayne Clarke, Uday S. Evani, Charles Markello, Kishwar Shafin, Xin Zhou, Arend Sidow, Vikas Bansal, Peter Ebert, Tobias Marschall, Peter Lansdorp, Vincent Hanlon, Carl-Adam Mattsson, Alvaro Martinez Barrio, Ian T. Fiddes, Chunlin Xiao, Arkarachai Fungtammasan, Chen-Shan Chin, Aaron M. Wenger, William J. Rowell, Fritz J. Sedlazeck, Andrew Carroll, Marc Salit, and Justin M. Zook. Benchmarking challenging small variants with linked and long reads. *Cell Genomics*, 2(5):100128, 2022.

[10] Takeo Yamada and Harunobu Kinoshita. Finding all the negative cycles in a directed graph. *Discrete Applied Mathematics*, 118(3):279–291, 2002.
