# Supplementary material for: JTK: targeted diploid genome assembler
Source: Bioinformatics. 2023 Jun 24;39(7):btad398. doi: 10.1093/bioinformatics/btad398 (PMC10320103; doi:10.1093/bioinformatics/btad398)
Supplement: btad398_Supplementary_Data [file btad398_supplementary_data.zip › jtk_suppl_fig.pdf]

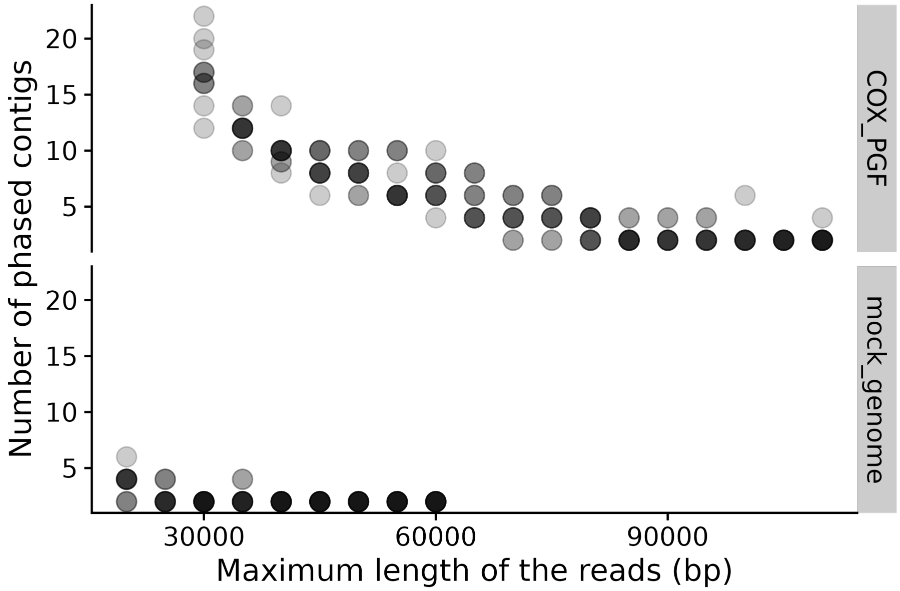


Figure S1: Relationship between the maximum length of the reads and the number of phased contigs. The number of phased contigs is two in the perfectly phased assembly (one contig per haplotype). Upper: The result obtained when the ground truth genome is COX and PGF haplotypes in the MHC region in the HG38 reference genome. Lower: The result obtained when the ground truth genome is synthetic genomes used in the main text.

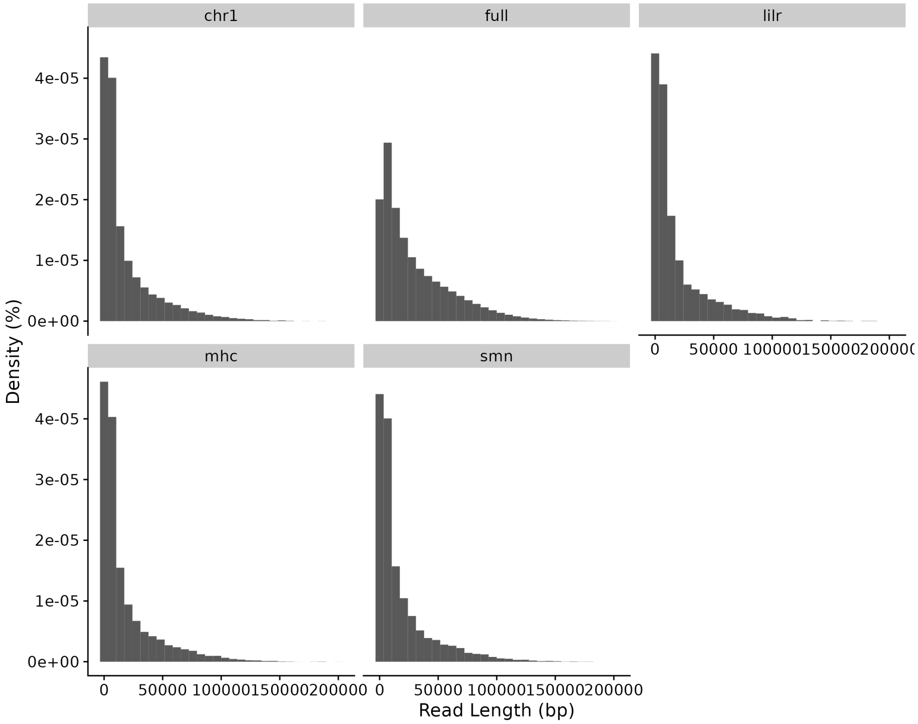


Figure S2: Histogram of read length on HG002. “mhc”, “chr1” and “lilr” represent the subsampled datasets used in this study, and “full” represents the histogram of the entire datasets. For visualization prepose, we set the cutoff at 200K bp.

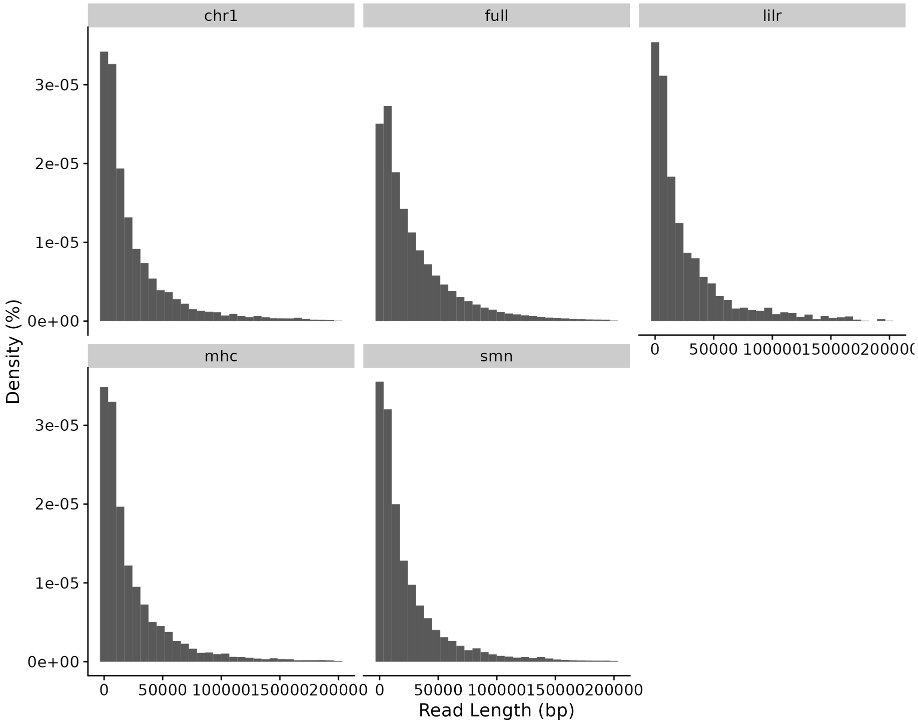


Figure S3: Histogram of read length on B080. The captions are the same as Fig. S2.

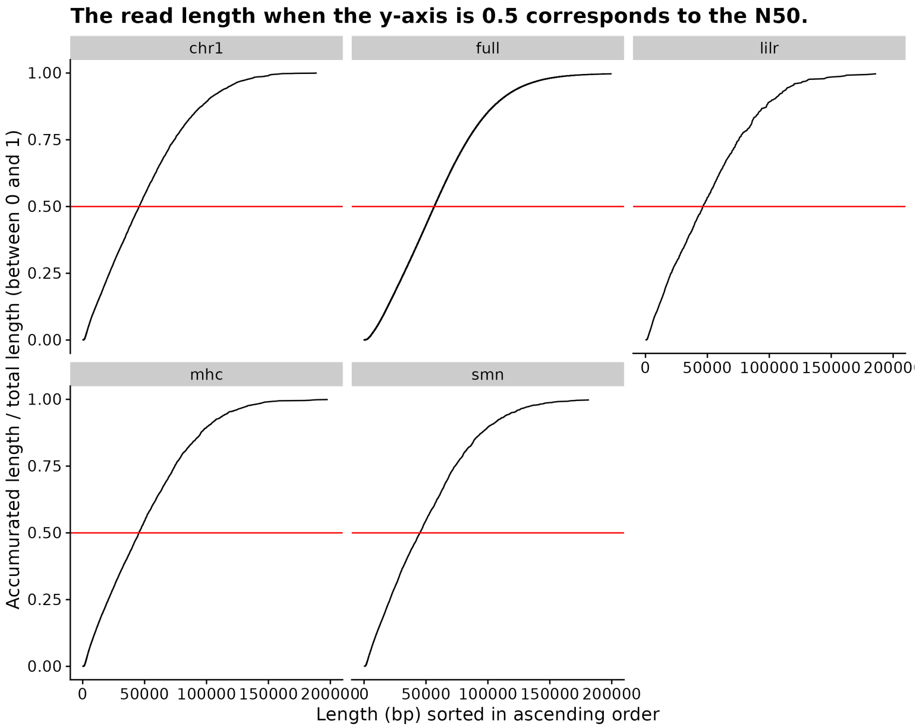


Figure S4: Relationship between cumulative lengths and sorted read lengths on hg002. Specifically, we first sorted the read lengths as $r_1 \leq r_2 \cdots r_N$, took the sum $R = \sum_i r_i$, and then plotted $(r_i, \sum_{k=1}^{i} r_k/R)$. "mhc", "chr1" and "lilr" subfigure represent the subsampled datasets used in this study, and "full" represents the histogram of the entire datasets.

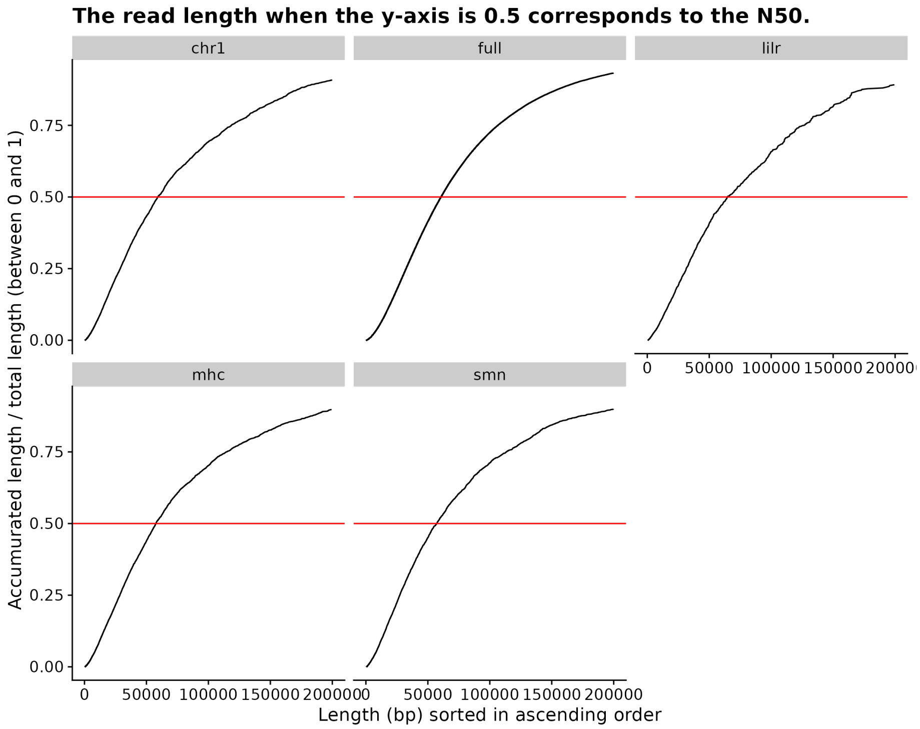


Figure S5: Relationship between cumulative lengths and sorted read lengths on b080. Specifically, we first sorted the read lengths as $r_1 \leq r_2 \cdots r_N$, took the sum $R = \sum_i r_i$, and then plotted $(r_i, \sum_{k=1}^{i} r_k / R)$. "mhc", "chr1" and "lilr" subfigure represent the subsampled datasets used in this study, and "full" represents the histogram of the entire datasets.

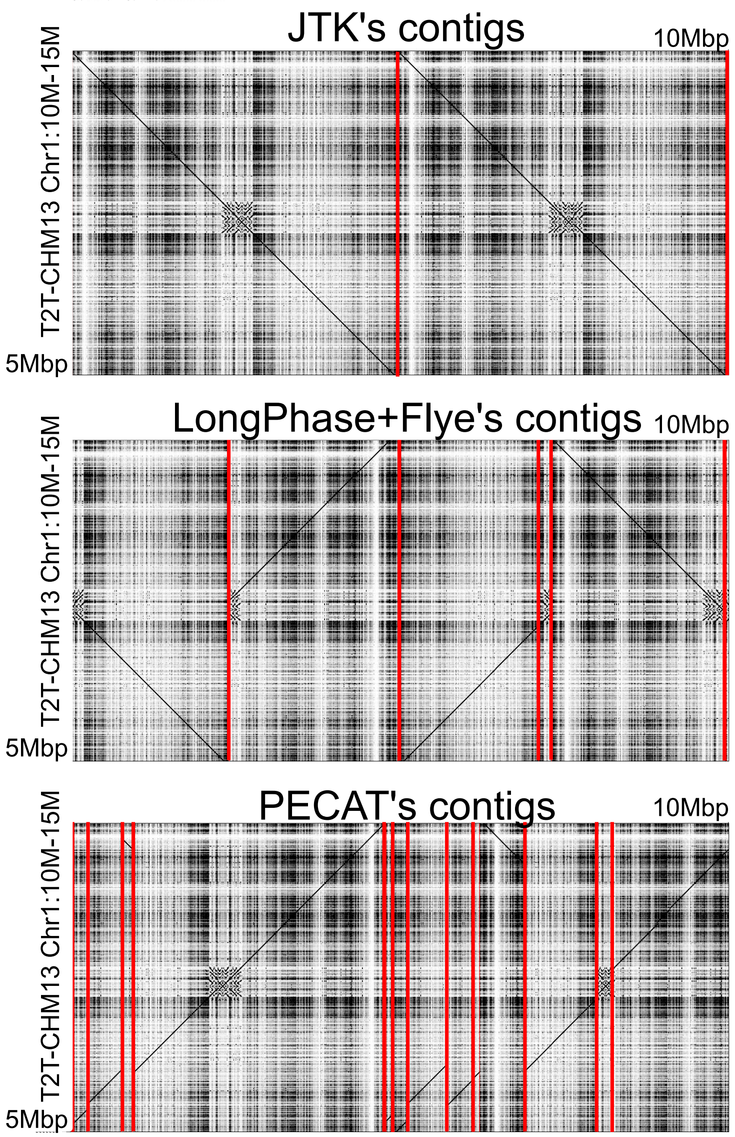


Figure S6: The dotplots between the assemblies in HG002 and T2T-CHM13 chr1:10M-15M. The red lines indicate the boundaries of contigs.

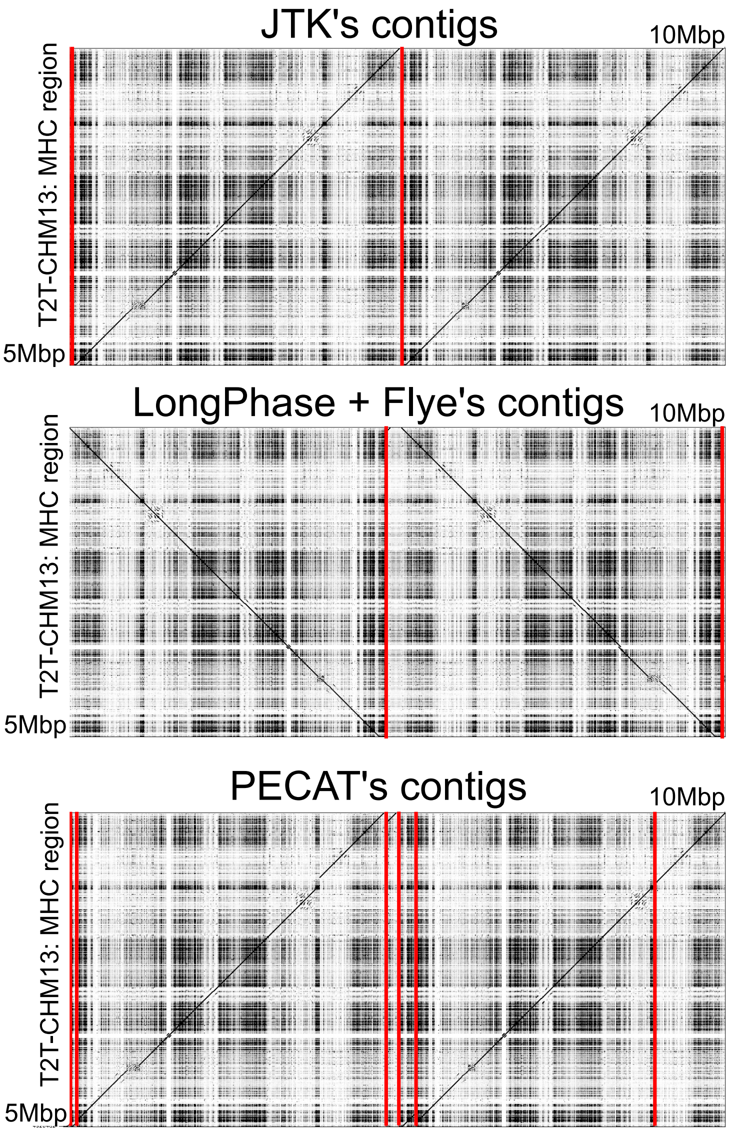


Figure S7: The dotplots between the assemblies in HG002 and the MHC region in the T2T-CHM13. The red lines indicate the boundaries of contigs.

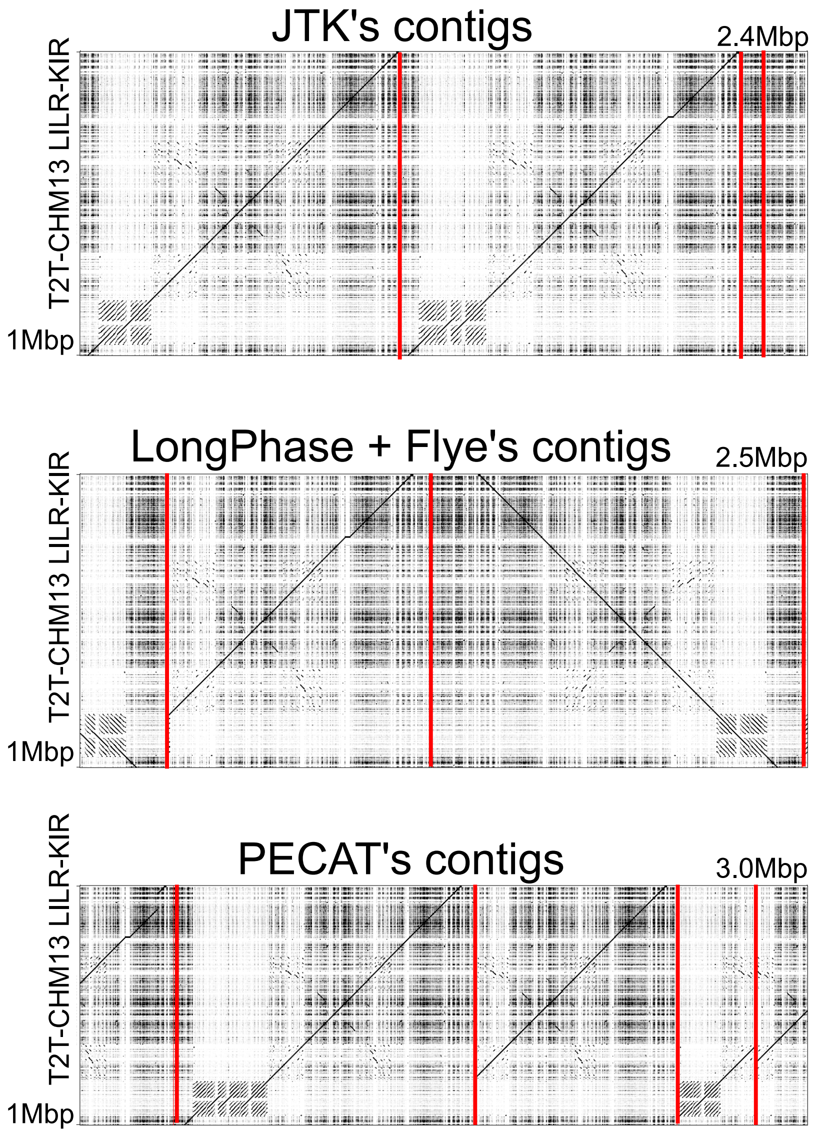


Figure S8: The dotplots between the assemblies in HG002 and the LILR-KIR region in the T2T-CHM13. The red lines indicate the boundaries of contigs.

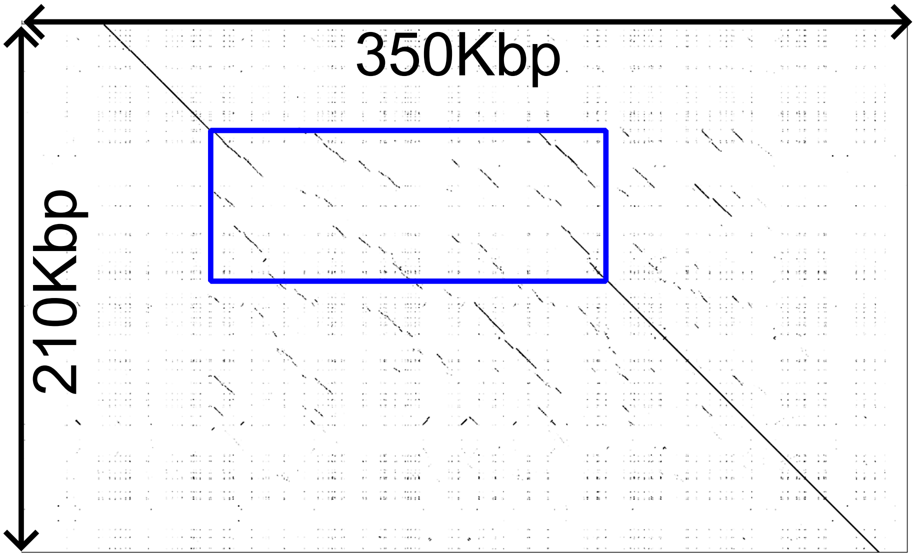


Figure S9: Dotplot of the highly variable region we explained in the main text in the MHC region in **HG002** assembled by `JTK`. The blue rectangle indicates the region we searched for annotations of genes.

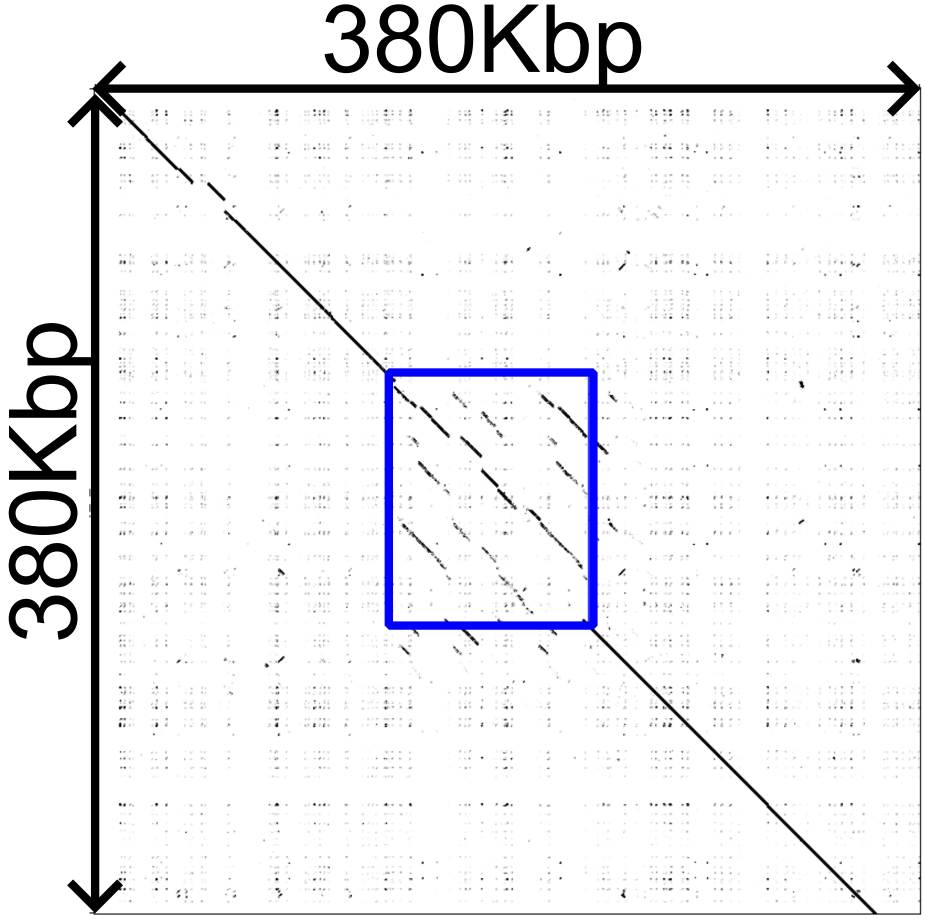


Figure S10: Dotplot of the highly variable region we explained in the main text in the MHC region in **B080** assembled by `JTK`. The blue rectangle indicates the region we searched for annotations of genes.

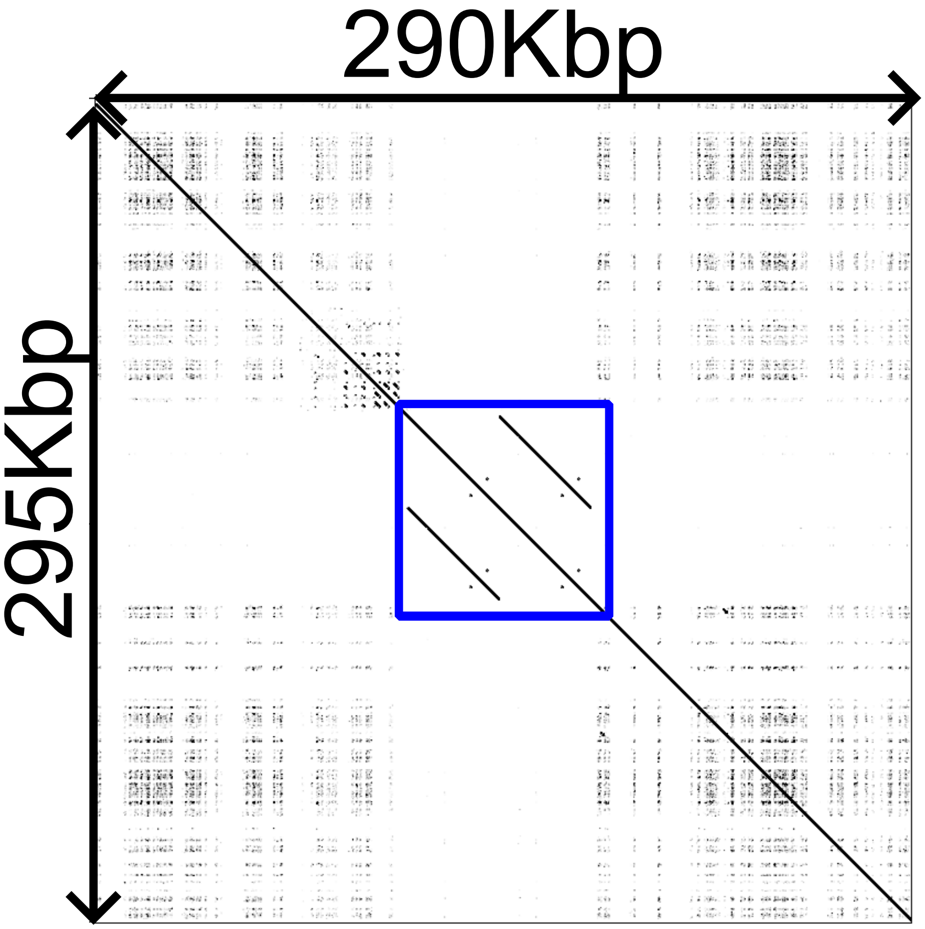


Figure S11: Dotplot of the segmental duplication in MHC class III region we explained in the **HG002** assembled by `JTK`. The blue rectangle indicates the region we searched for annotations of genes.

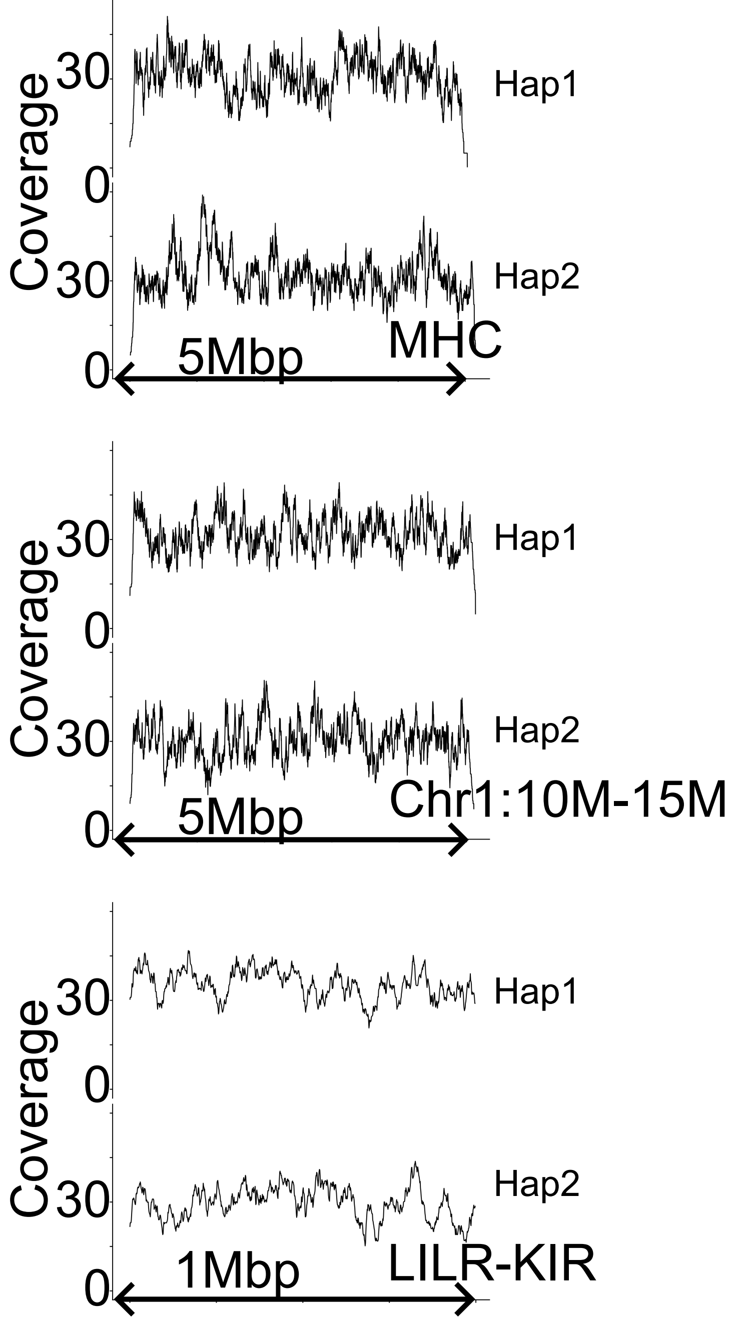


Figure S12: Coverages on the JTK's contig in B080.

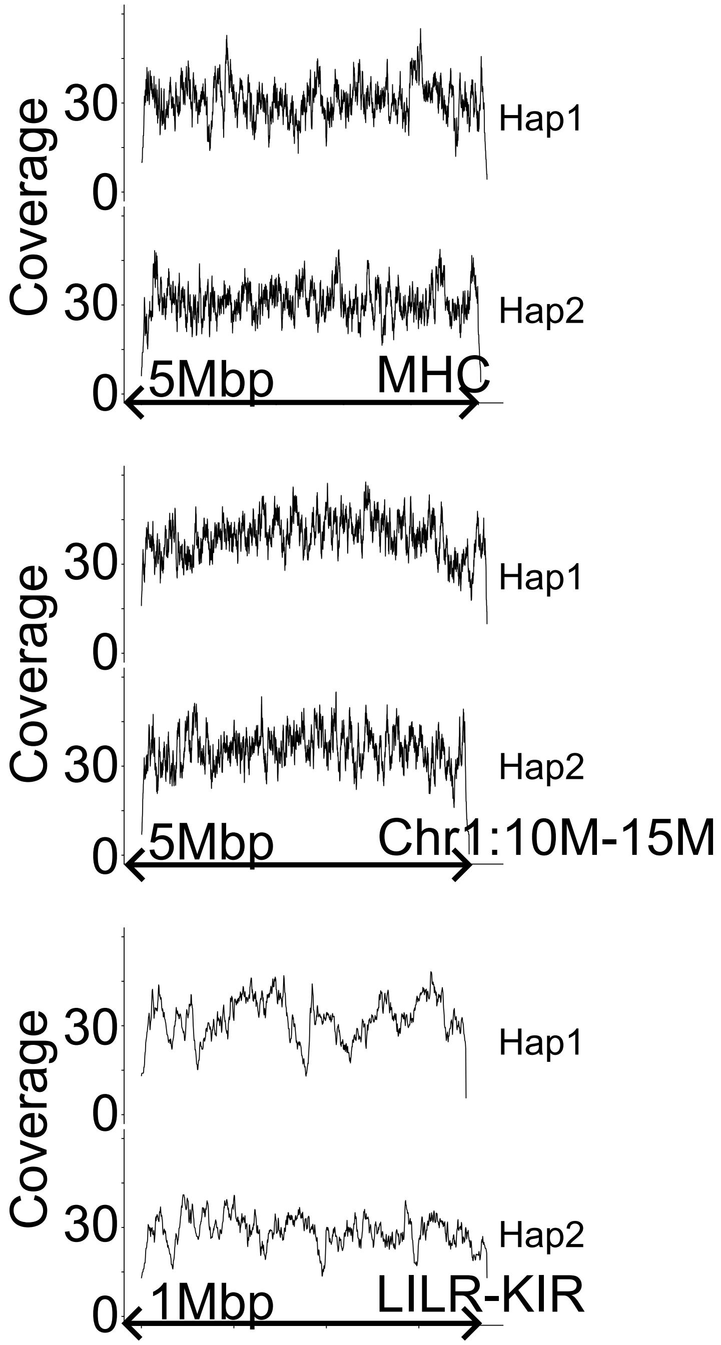


Figure S13: Coverages on the JTK's contig in HG002.

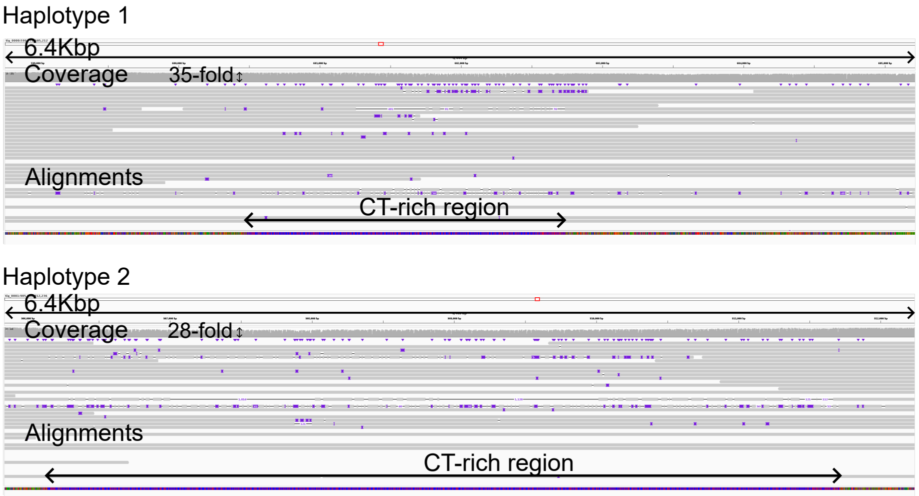


Figure S14: Alignments of ONT reads to JTK's assemblies. This region contains a large (~ 3Kbp) expansion of a low-complexity region consisting of C and T.

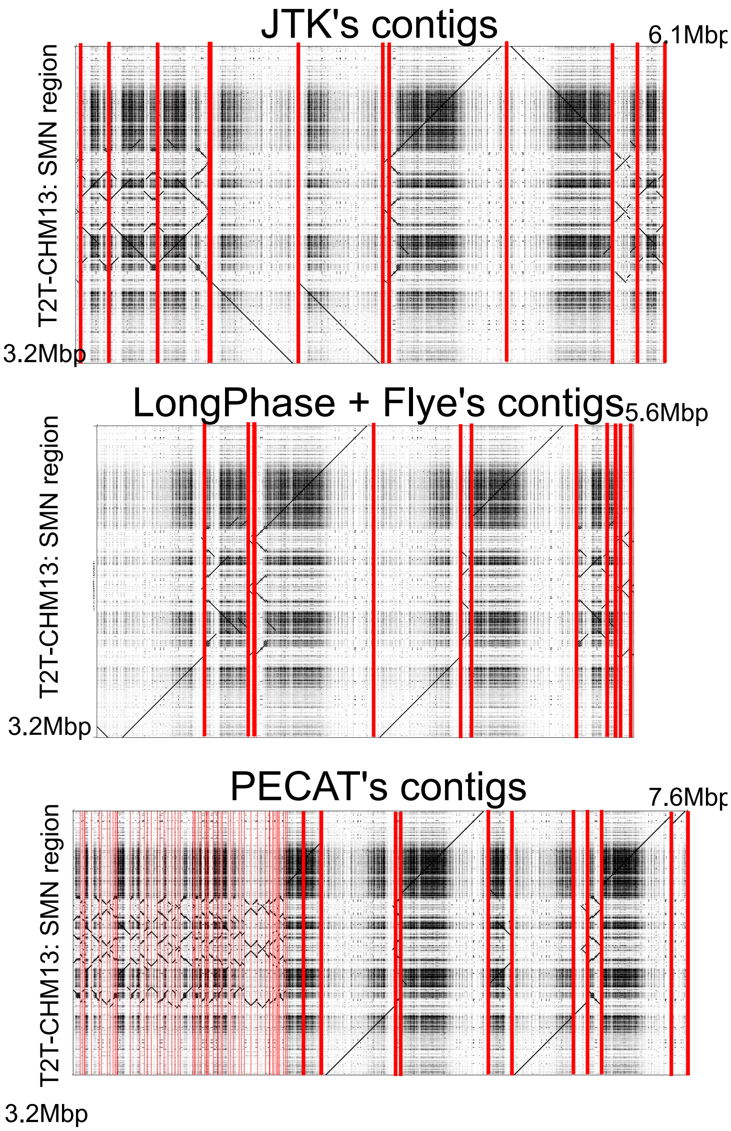


Figure S15: The dotplots between the assemblies in HG002 and the SMN region in the T2T-CHM13. The red lines indicate the boundaries of contigs.

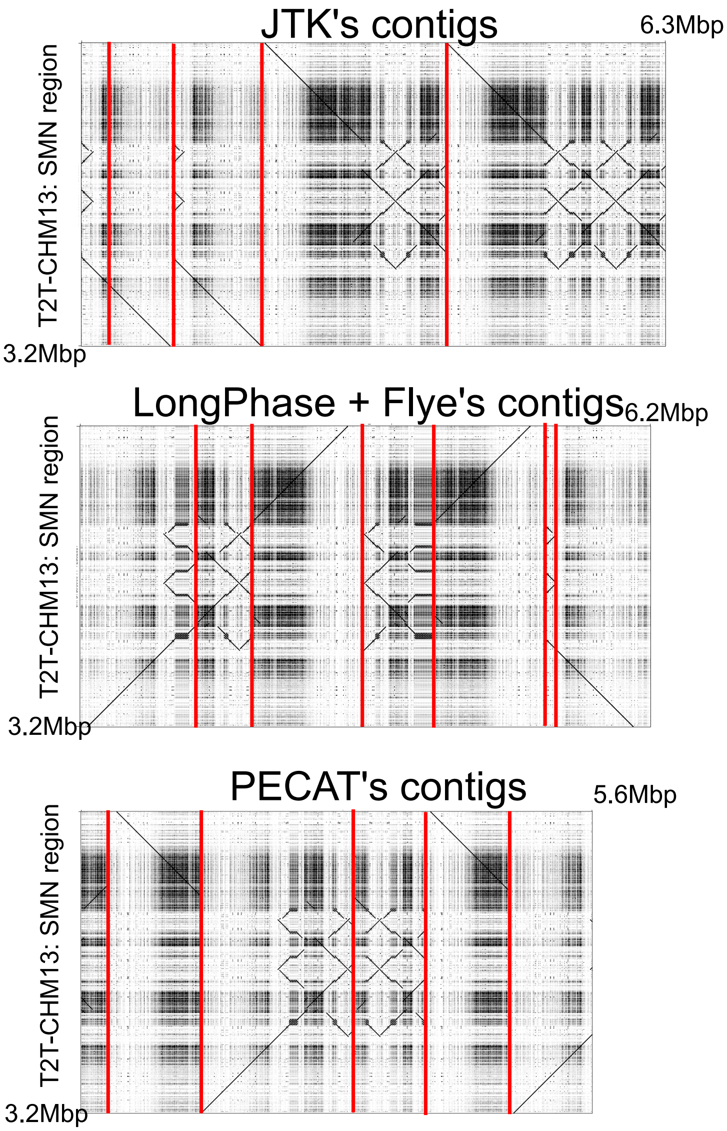


Figure S16: The dotplots between the assemblies in B080 and the SMN region in the T2T-CHM13. The red lines indicate the boundaries of contigs.
